# Testing the Stochastic Convergence of Ecological Indicators in BRICS in the Perspective of Public Health

**DOI:** 10.3389/fpubh.2022.897459

**Published:** 2022-04-29

**Authors:** Jinshun Wu

**Affiliations:** Department of Economics, School of Economics and Management, East China Jiaotong University, Nanchang, China

**Keywords:** BRICS, ecological indicator, fractionally integrated processes, stochastic convergence, structural break, C12, C14, Q50, Q54

## Abstract

This paper assesses the stochastic convergence of relative per capita ecological footprints within BRICS countries over the period 1961–2017 in the field of Public Health. Our initiatives have targeted ecological Indicator and health behaviors. Using the local Whittle estimator and some of its variants we assess whether relative per capita ecological footprints are long memory processes which, although highly persistent, may revert to their mean/trend in the long run thereby indicating evidence of stochastic convergence, or divergent processes in nutshell. Furthermore, we test whether (possibly) slow convergence or the complete lack of it may be the result of structural changes to the deterministics of each of the relative per capita footprint series by means of the tests of Berkes et al. (1) and Mayoral (2). For the ease of comparison, this paper assesses the stochastic convergence of relative per capita ecological capacities for BRICS as well. Our results show relatively strong evidences against stochastic convergence of ecological footprints. Furthermore, with regard to China and Russia, our results also decisively indicate that a slow or lack of convergence is the results of a structural break in the relative per capita ecological footprint series. However, our empirical researches support stochastic convergence of relative per capita ecological capacities for BRICS. In addition, we can conclude whether the per capita ecological footprints converge or not is dependent on the level of economic development, and the stochastic convergence occurs in those rich countries more probably, indicating that public health is becoming a more serious concern in developing countries.

## Introduction

The convergence implication envisaged by the neoclassical growth model is that if each country has the same savings rate, population growth rate and technological progress rate, the income level gap between countries will gradually disappear. Since the production of most goods and services requires the consumption of energies such as coal, natural gas and oil, energy-producing requires the exploitation of natural resources. The availability of many natural resources at low cost increases the vulnerability of countries' economic development models. Considering the close relationship between economic growth and environmental pollution, Grossman and Krueger (3) first introduced the Environmental Kuznets Curve (EKC). The EKC indicates that the degree of environmental deterioration will increase with the increase of the per capita income level, but the environmental quality will start to increase when the per capita income level reaches a certain threshold. This hypothesis means that there is an inverted U-shaped relationship between per capita income and environmental impact indicators and economic growth. At the same time, the view that economic growth will promote environmental improvement after a certain critical point has been discussed in many studies. Many studies believe that the income level of developing countries has not reached a critical point. With an increase in incomes, environmental pollution will continue to rise. On the other hand, developed countries are more successful in reducing environmental pollution, as their higher income levels may exceed the critical point or adopt cleaner energy technologies. Brock and Taylor (4) linked the environmental pollution indicators with the EKC hypothesis. Their Catch-up hypothesis means that poor countries have more environmental pollution. Moreover, in the initial stage, the gap of environment indicators between the rich and poor countries is divergent. This difference is rooted in the difference in the initial capital stock of the two countries. However, with their incomes increasing, once the developing countries start to use the advanced technology of environmental protection, the difference in environmental quality between the two countries will be reduced and various environmental pollution indicators will converge. Although the initial stage of economic growth resulted in environmental damage, the environmental quality is bound to be improved eventually.

In the past, air pollutants were used as a single indicator to measure environmental quality, which could not fully reflect the improvement of environmental quality. Now there is a more comprehensive environmental indicator, the ecological footprint. Ress (5) first proposed the concept of ecological footprint (EF), which was then developed by 4. EF is a measure of human demand for natural resources and services, revealing the relationship between human lifestyle, consumption patterns and consumed natural capital (5), which can comprehensively evaluate the feasibility of achieving a country's sustainable development goals. The Global Ecological Footprint Network describes the ecological footprint as: the ecological footprint measures the amount of “biologically productive” land or water that enables human beings to sustain itself. The term “biologically productive areas” refers to the areas with biological productivity that are needed to maintain the survival of individuals, regions or countries, or that can absorb wastes discharged by human beings. The ecological footprint can be used to evaluate and manage resource use throughout the country, measure and assess if people's lifestyles are sustainable, and account how much waste can be absorbed by the ecosystem, also known as “appropriated carrying capacity.” EF consists of 6 sub-footprints: carbon footprint, fishing grounds footprint, forest footprint, cropland footprint, built-up land footprint and grazing land footprint. These ecological footprints reflect a country's ownership of natural resources and the ecologically productive area that has been used, and can comprehensively reflect the environmental problems as well. Moreover, the ecological footprint is a resource accounting tool that measures how much renewable energies in the biosphere can be used by human activities. In addition, EF can also measure the ecological cost of goods and services that the nature provides to humans, such as land, and the maximum sustainable population in a given area. In a word, EF indicates the environmental limit and the extent to which human beings exceed the natural limit, which can assist countries to evaluate how the ecological resources are being used.

This study focuses on the BRICS countries for the following reasons:

First, compared with many other emerging countries, the BRICS countries have experienced a rapid transition from an ecological surplus to an ecological deficit. This is mainly due to the rapid economic growth of these countries over the past decade. The BRICS countries contribute 21% of global GDP and have 41% of the world's population. These countries have an average annual economic growth rate of 6.5% (6). Moreover, between 2005 and 2016, the total GDP of the BRICS countries ranged from US$ 218.7 billion to US$ 162.6 billion. The rapid economic growth has also led these countries to consume more than 40% of the global total energies, and they are also the major global CO_2_ emitters (7). They are facing both environmental and natural resource stresses due to their rapid economic growth. Their ecological indicators may be long-memory and may not converge. In view of this, in order to reverse the growing trend of carbon dioxide emissions, reasonable policies are needed to ensure that while economic development is taking place, it will not cause huge damage to the ecological environment.

Secondly, despite the huge potentials of renewable energies, BRICS countries still rely excessively on fossil fuels to meet their energy needs, which poses a huge threat to the environment. At the same time, while pursuing high-speed economic development, the region has also experienced a continuous decline in ecological sustainability. In addition, the research on this area can help the policy makers to prevent the environmental deterioration with practical and reasonable policies.

## Literature Review

The literature on the deterioration of ecological footprints mainly focuses on developed countries and international organizations mainly composed of developed countries. As far as we know, the first paper on convergence of ecological footprints is Ulucak and Lin (8). They analyzed the random behavior of ecological footprints and each sub-component using Fourier unit root test *via* the dataset of ecological footprints of each state in the United States from 1961 to 2012. The empirical results show that the ecological footprints of states in US are non-stationary, and not mean-reverting processes. Therefore, an impact on these sequences will result in a permanent deviation from the long-term equilibrium paths. Since then, there have been a lot of relevant researches mainly exploring developed countries' ecological indicators. For example, Ulucak and Apergis (9) studied the convergence of the per capita ecological footprints using data samples from 20 EU countries during the period 1961–2013. The results show that there are three convergence clubs. Bilgili and Ulucak (10) studied the convergence of per capita ecological footprints among G20 countries during 1961–2014 by using bootstrap panel KPSS unit root test and club convergence test considering structural breakpoints. It is concluded that the per capita ecological footprints of these countries will reach random convergence and deterministic convergence, and two convergence clubs are identified. Solarin (11) uses LM and RALS-LM unit root methods to test the stochastic convergence of ecological footprints of 27 OECD countries from 1961 to 2013. They found that the ecological footprints and carbon footprints of 25 countries are stationary. They also performed β convergence and σ convergence test in their research. Among the 25 countries with random convergence, the ecological footprints of 13 countries are β convergent; among them, 15 countries have β convergent carbon footprints. In terms of σ convergence, the ecological footprints and carbon footprints of all countries selected meet the convergence criteria. Studies show that, although not all countries meet the convergence conditions, those countries in which convergence happened already can jointly formulate environmental protection policies to improve convergence among more countries. Yilanci et al. (12) uses the annual data of ecological footprints and each sub-component of 25 OECD countries from 1961 to 2013, and uses various stability test methods to prove that the other footprint sequences except the fishing ground footprint are stationary. Yildirim et al. (13) proposed the Fourier cross-section panel KSS unit root test to study the convergence of ecological footprints among 16 EU membership countries. They further use the rolling window method to consider the time-varying stationarity of the ecological footprint sequences. Through their study on the convergence of the ecological footprints and each sub-component, they prove that the convergence or divergence of the ecological footprints has different states in different periods.

In recent years, there have been a large number of researches on the convergence of ecological footprints between countries with different levels of development, and the relationship between the convergence of ecological footprints and the degree of economic development is discussed. Solarin and Bello (14) studied the convergence of the ecological footprints of 128 countries with different levels of development during the period 1961–2013. The results show that the ecological footprint series of 96 countries (81%) are non-stationary and there is no mean reversion (non-convergence). This means that various environmental protection policies have long-term and permanent effects in many countries. Bilgili et al. (15) selected 15 countries in Asia, Africa, America and Europe to test the stationarity of the ecological footprints, and asserted that the ecological footprints of the Asian group are non-stationary and diverge. Using the club convergence method proposed by Phillips and Sul (16) and Solarin et al. (17) showed that 92 countries' ecological footprints and each sub-component have club convergence. Ozcan et al. (18) used panel KSS and Fourier panel KSS to study the stability and convergence of ecological footprints in 113 countries with different levels of development depending on their national development levels. The ecological footprints of all high-income countries, some low-income countries and upper-middle-income countries are stable, while the ecological footprints of lower-middle-income countries are not stationary. Erdogan and Okumus (19) used the annual sample data from 1961 to 2016 to test the random convergence of ecological footprints and club convergence of different income groups. The FPKPSS panel statistics of the countries in the high, middle and low income groups indicate that the per capita ecological footprints are non-stationary. At the same time, the FKPSS method was also used to test the stability of ecological footprints at the national level. On this occasion, 6 out of 26 high-income countries and 8 out of 38 middle-income countries have stationary ecological footprints. In 8 out of 25 low-income countries ecological footprints are stationary. At the same time, the club convergence method was used to test the convergence of the ecological footprints. It is concluded that there are convergence clubs in different income groups. Işik et al. (20) employed the threshold autoregressive panel unit root test to study the convergence of ecological footprints of NAFTA countries during 1961–2016. The study suggested that EF converges at the latter stage of the threshold, and the second stage accounts for 48.8% of the total sample. The ecological footprints of the first stage are divergent. These conclusions highlight the need for NAFTA countries to formulate common environmental policies to mitigate environmental degradation.

With the rapid economic development in developing countries, the academic community has become more and more interested in the convergence of the ecological footprints of international organizations composed of developing counties in recent years. Yilanci and Pata (21) used the panel data of the five ASEAN countries' per capita ecological footprints from 1961 to 2016, and used two-mechanism threshold autoregressive panel unit root test to study the convergence of ecological footprints. Researches argue that the ecological footprints in the second mechanism are divergent; in the first mechanism they are absolutely convergent. The second mechanism accounts for 80% of the sample, so the results strongly support that the per capita ecological footprints of the five ASEAN countries are absolutely convergent, and they believe that common policies should be implemented to slow down the environmental deterioration. Tillaguango et al. (22) studied the convergence of the ecological footprints of Latin American countries from 1990 to 2016 and found that there exits a per capita ecological footprint club. Using the method of logistic regression, the paper explored the factors that affect the convergence of the ecological footprint clubs and found that economic complexity, shadow economy and abundance of natural resources can significantly affect the formation of the clubs. Their research indicated that there are three convergence clubs in Latin America, and the two clubs are divergent.

As can be seen from the literature review above, a large number of techniques have been applied to test the stochastic convergence and divergence of ecological and environmental indicators (8, 10, 13, 18, 20, 23, 24), but most of these studies only used the time series econometric method to analyze the existence of its stochastic convergence characteristics. From a random point of view, if the per capita ecological footprint sequences tend to converge as time lapses, the impacts of external shocks on the sequence should be only temporary, and the data are stationary. In the presence of a unit root, the external impacts on the sequence will be permanent, indicating that these sequences are divergent. From the literature review, it can also be clearly seen that the evidence of convergence of ecological and environmental indicators is very complicated. One possible explanation is that the existing literature ignores the possibility that ecological footprints are long memory processes, i.e., they are mean- (or trend-) reverting, but it will take a long time for these processes to arrive at their means or trends. There are two reasons to doubt this view: First, a country's ecological footprints are determinated by a lot of fundamental factors, such as economic size, economic structure and technological level. All these factors may only change slowly as time goes by, so environmental indicators such as relative ecological footprints between countries may also change slowly. Secondly, previous studies have found that GNP and per capita GNP are long memory processes (2). The long memory process means that there exists significant interdependent relationship between two observations over a long time, so the effects of shocks tend to decay slowly, although they are still mean-reverting in nature. If environmental indicators such as ecological footprints are indeed long memory processes, then previous studies might provide misleading results. For example, if the null hypothesis of a unit root has not been rejected in previous studies, this may not mean a lack of convergence, but may reflect that the environmental indicator has the difference order of fractionally integrated processes, which might not be integer. Therefore, this means that the convergence process is taking place, albeit vary slowly.

The analysis in this paper focuses on conditionally stochastic convergence, which does not require every country (region) to converge to the same steady-state level just as β and σ convergence do. When the per capita ecological footprint gap between countries (regions) follows a mean stationary process, i.e., the impact of the relative per capita ecological footprints only brings temporary deviation from their convergent trends. This paper uses the stochastic convergence test approach to determine whether it can be ensured that ecological indicators converge permanently in a country. If such a necessary mechanism does not exist, deviations from trends are permanent. Thus, the concept of convergence is more important to the study of ecological footprints because it can test the durability of impact effects. In this regard, few researchers use unit root test to study whether the impacts on ecological indicators are temporary or permanent. The lack of convergence researches makes it difficult for a country to take joint actions to hinder environmental deterioration. The stochastic behavior and dynamic changes of ecological indicators will help to formulate sustainable development policies. If ecological indicators are stationary, their impacts exerted by external shocks are transient, and these sequences will converge stochastically to their means once these impacts disappear. On the contrary, if the impacts are permanent, the sequences will diverge from their means. If the evolution trend of variables is known, the convergence and divergence of environmental indicators make it easier for policymakers to apply more effective environmental protection policies. In this paper, the fractionally integrated autoregressive moving average model (ARFIMA) is used to estimate the fractionally integrated parameters of the ecological indicators using the 1961–2017 data set. The ARFIMA model is a generalized version of ARIMA and an improvement on the *I*(0)|*I*(1) dichotomy. It can estimate the integration order parameters, which can be of any real number in the data generated processes. Because the unit root test can only judge whether the ecological indicators are convergent I (0) processes or divergent I (1) processes, and cannot test the convergence speed, the fractionally integrated model may be more appropriate.

In this paper, for exploring whether the relative ecological indicators per capita contain unit roots (the null hypothesis is a non-convergent process). We leverage the local Whittle (LW) estimation method (25) and its modified versions, e.g., exact local Whittle (ELW) and two-stage detrended (breakpoint) exact local (ELW) estimation method (26, 27) to estimate the parameters of fractionally integrated model. In contrast, the alternative assumption is that they are long-term memory processes that, although highly persistent, may return to their mean/trend over the long term (meaning slow convergence). The existence of structural breakpoints may lead to biased estimation of parameters [see (28, 29)]. In the case of the fractionally integrated processes, this problem is mostly applied to evaluate their expectation performance. From our point of view, there is evidence that the existence of structural breakpoints on the deterministic trend may lead to an overestimation of the order of the fractionally integrated process, so the conclusions drawn are biased in favor of supporting the long memory process [see (30, 31)]. In this study, the structural breakpoints are incorporated into the ARFIMA model by introducing dummy variables into the deterministic components *Z*_*t*_ of Equation (5), including mean breakpoints and trend breakpoints, respectively. Furthermore, the Mayoral (2) test is used to identify the false long memory state in the ecological indicator sequences, i.e., the long memory phenomenon is caused by the existence of breakpoints in the deterministic trend of the sequences. Finally, we use the non-parametric CUSUM statistics of Berkes et al. (1) to determine the points in time when the structural changes of the ecological indicator sequences occur (if there is a breakpoint).

## Model

In this paper, the fractionally integrated models are used to test whether the environmental indicators, e.g., ecological footprints and ecological carrying capacities in BRICS converge. The idea of this test is that if the per capita environmental indicators are converging between countries, the logarithm of the ratio of the per capita environmental indicators in a country to the average environmental indicators in all countries should be stationary, or at least the mean- (or trend-) reverting. Therefore, referring to Strazicich and List (32), this paper defines *y*_*it*_ as the natural logarithm value of the relative environmental indicators per capita in *t* year of the *i* country, and its expression is shown in Equation (1):

(1) $$\begin{array}{l} y_{it}=\ln\;\left(\frac{PCE_{it}}{J^{-1}\overset{J}{\underset{j=1}{\sum }}PCE_{it}}\right) \end{array}$$

The *PCE*_*it*_ is the per capita environmental indicator in *t* year for the *i* country. *J* is the total number of countries. In order to test the null hypothesis that per capita environmental indicators diverge, we examine whether the natural logarithm *y*_*it*_ of the annual per capita relative environmental indicators in the *i* country contain a unit root, or whether they can be characterized by trend-stationary and mean-reverting processes.

### Discrimination and Test of Convergence of Relative Environmental Indicators per Capita

When a time series has the structural change in its deterministic trend, the standard unit root test method often has the problem of significant level distortion; Moreover, when the unit root of the sequence is close to 1, the performance of standard unit root test is affected substantially. In other words, the unit root test is often unable to decide whether a sequence is highly persistent or infinitely persistent. The test method based on fractionally integrated processes provides a general framework, which can test whether an ecological and environmental indicator is an I (1) process with infinite persistence or with long persistence (which may be not covariance-stationary), but characterized by mean reversion in the long term, indicating that the environmental indicator is an *I*(*d*) process with the integration order bounded the domain that 0 < *d* < 1. The fractionally integrated expression of a environmental indicator sequence containing a time trend, such as the ARIMA (0, d, 0) model, is shown by Equation (2):

(2) $$\begin{array}{l} y_{it}=c_{i0}+\gamma_{i}t+x_{it} \end{array}$$

(3) $$\begin{array}{l} {\left(1-L\right)}^{d_{i}}x_{it}=u_{it} \end{array}$$

where *u*_*it*_ is a white noise process with zero mean value, *d*_*i*_ is the integration order of a single sequence and a deterministic trend function (there may be breakpoints). According to various values of *d*_*i*_, we can identify the states of convergence or divergence of relative per capita environmental indicators.

Scenario 1: −0.5 < *d* ≤ 0. This is a short memory process, i.e., there is “fast convergence” or “short memory convergence.”
Scenario 2: 0 < *d* < 0.5. This is a long memory but still stationary process, and also a convergent process with slowly (or smoothly) decaying speed. In this case, a country's environmental indicators might undergo a long period of convergence toward a common long-term trend.
Scenario 3: 0.5 < *d* < 1. This is a long memory process, which is non-stationary but still has the property of mean reversion. In this case, the process is characterized by a high degree of persistence. Therefore, the impact of the distant past will still have lasting effects on the present.
Scenario 4: *d* ≥ 1. This is a unit root or divergent process. In this case, any initial shock has a significant impact on the future, and the process is unlikely to return to the mean at some point in the future.

The testing approach to the fractionally integrated order allows a richer classification of convergence states, so that stationary convergence and non-stationary convergence but with mean reversion can be distinguished. Another characteristic of this classification scheme is that it can take into account the lasting impact of the previously invoked shock on the present, or the rapid attenuation without any impact on the present, and more importantly, it can take into account the states in between two extremes mentioned above. The simple *I*(0)|*I*(1) dichotomy could not capture these states because the standard unit root test can only considers two extreme cases, i.e., persistence or no persistence at all.

Then, we use local Whittle likelihood estimation method (LW) and various improved versions to estimate the parameter of fractional integration *d* of Equation (2). The advantage of this method is that the estimator is not affected by the non-stationarity of the short-term process. In order to judge whether a per capita environmental indicator is an unit root process or a mean-reverting and long memory process with finite or inflated variance (depending on the estimated $\hat{d}$ value), we conduct hypothesis test of the null hypothesis $H_{0}:\hat{d}=d_{0}$ and the alternative hypothesis $H_{1}:\hat{d}\neq d_{0}$. If the hypothesis holds that per capita relative environmental indicators are mean-reverting, there is enough evidence to prove that these indicators are stochastically convergent processes.

After applying LW, ELW, two-stage detrended ELW (2ELWd) and the 2ELWd method with breakpoints removed to obtain an estimate of the order of the fractional integration $\hat{d}$ of the individual environmental indicator series, we use the Robinson (33)'s LM test framework to test the hypothesis of the environmental indicator series of each country as shown in Equation (4):

(4) $$\begin{array}{l} H_{0}:d=d_{0} \end{array}$$

In this hypothesis test, the null hypothesis is the existence of unit root. The alternative is that the per capita environmental indicators have long memory characteristics. Robinson (33) proposed the following statistics and proved that the standard normal distribution is satisfied under certain regularity conditions:


(5) $$\begin{array}{l} \hat{R}={\left(\begin{array}{c} \frac{n}{\hat{A}} \end{array}\right)}^{1/2}\frac{\hat{a}}{{\hat{\sigma }}^{2}}\sim^{d}N\left(0,1\right),n\to \infty \end{array}$$

where,


(6) $$\begin{array}{l} \hat{A}=\frac{2}{n}[\overset{n-1}{\underset{j=1}{\sum }}\psi {\left(\lambda_{j}\right)}^{2}-\overset{n-1}{\underset{j=1}{\sum }}{\psi \left(\lambda_{j}\right)\hat{\varepsilon }\left(\lambda_{j}\right)}^{\prime }\times {\left(\overset{n-1}{\underset{j=1}{\sum }}\hat{\varepsilon }\left(\lambda_{j}\right)\hat{\varepsilon }{\left(\lambda_{j}\right)}^{\prime }\right)}^{-1}\\ \;\times \overset{n-1}{\underset{j=1}{\sum }}\hat{\varepsilon }\left(\lambda_{j}\right)\hat{\varepsilon }{\left(\lambda_{j}\right)}^{\prime }] \end{array}$$

(7) $$\begin{array}{l} \hat{a}=\frac{-2\pi }{n}\overset{n-1}{\underset{j=1}{\sum }}\psi \left(\lambda_{j}\right)g{\left(\lambda_{j},\hat{\tau }\right)}^{-1}I\left(\lambda_{j}\right) \end{array}$$

$$\begin{array}{l} \psi \left(\lambda_{j}\right)\text{=}\log\left(2\sin\frac{\lambda_{j}}{2}\right);\hat{\varepsilon }\left(\lambda_{j}\right)\text{=}\frac{\partial }{\partial \tau }\left[\log g\left(\lambda_{j};\hat{\tau }\right)\right];\;\lambda_{j}=\frac{2\pi j}{n}. \end{array}$$

*I*(λ_*j*_) represents the periodogram of $\hat{u}$_*t*_ as follows:

(8) $$\begin{array}{l} I\left(\lambda_{j}\right)=\frac{1}{2\pi n}{\left|\overset{n}{\underset{t=1}{\sum }}{\hat{u}}_{t}e^{i\lambda_{j}t}\right|}^{2} \end{array}$$

where $\hat{u}$_*t*_ is the least squares residual obtained from Equation (2), *g* is a known function from the spectral density:

(9) $$\begin{array}{l} f\left(\lambda_{j};\tau \right)=\frac{\sigma^{2}}{2\pi }g\left(\lambda_{j};\tau \right) \end{array}$$

Robinson (33)'s test framework relies on some specific assumptions imposed on the short memory component *u*_*t*_. If it is a white noise, *g* ≡ 1; If it is a process that follows as the form of AR (1) *u*_*t*_ = τ*u*_*t*−1_ + η_*t*_, $g\left(\lambda_{j};\tau \right)={\left|1-\tau e^{i\lambda_{j}}\right|}^{-2}$, and $\sigma^{2}\text{=var}\left(\eta_{t}\right)$. The parameter considered is the long memory parameter *d*, which shows whether a time series process is long memory. Because that *d* < 1 means the relative per capita environmental indicators converge, the null hypothesis is set as follows in this study:

(10) $$\begin{array}{l} H_{0}d=d_{0}=1. \end{array}$$

This study uses the data set of the BRICS countries from 1961 to 2017 for empirical analysis. Obviously, the data set is a small sample (*n* = 57), so it is necessary to calculate the empirical distribution of various local Whittle estimators. When the sample size *n* = 57 and the truncation parameter α is in the interval [0.65, 0.8], we will give the accurate distribution of the memory parameter *d* estimators of LW, ELW, 2LWD and the detrended 2LWD method, and then construct the confidence interval of the estimators.

### Convergence Pseudo-Long Memory Test of Relative Environmental Indicators per Capita

That relative environmental indicators are long memory processes indicates that it takes a long time for these indicators to approach convergent paths. However, although a time series (as mentioned above *y*_*it*_) is a long-term memory process, it is not clear whether it is indeed a long memory process or a short memory and stationary one with a mean shift (showing the characteristics of long-term memory processes). Although these two cases show that the sequences of relative environmental indicators per capita converge, it is slower for the stationary processes with mean shifts to approach convergence than the real long memory processes. If their means shift toward the upward direction of the sequences, these indicators converge more slowly; If means shift downwards, these sequences converges faster. After obtaining evidence that these indicators are long memory I (d) or infinite memory I (1) processes, this paper will continue to determine whether the potentially slow convergence or lack of convergence is real or the result of a structural change in the mean of an otherwise stationary process.

This paper introduces a test method proposed by Mayoral (2) to solve this spurious long memory problem. If per capita environmental indicators are pseudo-long memory processes, it means that these processes are stationary, but at a certain time point, a structural fracture occurs in the opposite direction of the sequence trend, indicating that the per capita environmental indicator would reach convergence after a shorter time. These two types of models fitting the two processes can be nested together and expressed as Equation (11) below. This paper assumes that the relative environmental indicator data set *y*_1_, *y*_2_, ···, *y*_*n*_ are generated from the model listed as follows:

(11) $$\begin{array}{l} y_{t}=\beta^{\prime }\cdot Z_{t}+\delta \cdot V_{t}\left(\omega \right)+x_{t} \end{array}$$

Where *Z*_*t*_ is a deterministic component, and *V*_*t*_(ω) can be a constant or a trend function. It is used to judge whether the time series has undergone structural changes. The observations of the time series are *Z*_*t*−*k*_ after the break point appears, while they are evaluated as zeros before the break point appears. Meanwhile, the random term *x*_*t*_ is defined as:

(12) $$\begin{array}{l} {\left(1-L\right)}^{d}x_{t}=u_{t},\begin{array}{c} \varepsilon_{t} \end{array}\sim N\left(0,\sigma^{2}\right) \end{array}$$

Based on this data-generating structure, the hypothesis testing should set the following null and alternative hypothesis:

(13) $$\begin{array}{l} \{\begin{array}{c} H_{0}:d\in D_{0},\begin{array}{c} \delta =0,D_{0}\subset \left(0.5,1.5\right) \end{array}\\ H_{1}:d=0,\begin{array}{cc} \delta & noconstraint \end{array} \end{array} \end{array}$$

To test the above hypotheses, Mayoral (2) proposed a semi-parametric test method, and constructed a Most Powerful Invariant (MPI) statistics by comparing the log-likelihood ratio under the null hypothesis *H*_0_ and the alternative one *H*_1_. This consistent statistics $R_{b}^{f}\left({\hat{d}}_{n}\right)$ is of the form as follows:

(14) $$\begin{array}{l} R_{b}^{f}\left({\hat{d}}_{n}\right)=\\ \;n^{1-2{\hat{d}}_{n}}{\left(\frac{{\hat{\lambda }}^{2}}{{\hat{\gamma }}_{0}}\right)}^{-1}\frac{\underset{\omega \in \Omega }{\inf}{\left(y-Z\cdot \tilde{\beta }-V\left(\omega \right)\tilde{\delta }\right)}^{\prime }\left(y-Z\cdot \tilde{\beta }-V\left(\omega \right)\tilde{\delta }\right)}{{\left(\Delta^{{\hat{d}}_{n}}y-\Delta^{{\hat{d}}_{n}}Z\cdot \hat{\beta }\right)}^{\prime }\left(\Delta^{{\hat{d}}_{n}}y-\Delta^{{\hat{d}}_{n}}Z\cdot \hat{\beta }\right)} \end{array}$$

where the order ${\hat{d}}_{n}$ of fractional integration is the uniform estimator obtained by ELW and other methods. As a semi-parametric test method, the statistics should be evaluated by resorting to the ordinary least square method (OLS) to estimate the parameters in Equation (11) under *H*_0_ or *H*_1_ hypothesis, respectively. All the parameters of the numerator, including $\tilde{\beta }$ and $\hat{\delta }$ on the right side of the Equation (14) are estimated when the null hypothesis *H*_0_ holds, while $\tilde{\beta }$ and $\hat{\delta }$ in the denominator are estimated under the alternative hypothesis *H*_1_. At the same time, under the null hypothesis *H*_0_, ${\hat{\gamma }}_{0}$ represents ε_*it*_ variance estimator. ω = *k*/*n* is the ratio of the number of sub-sample observations before the possible break point to the total sample. Considering that allowing the break point to appear in the whole interval (0, 1) will cause the test to have a very low power potentially, we adopt the restricted interval Ω = [0.15, 0.85] suggested by Andrews (34). In addition, the parameter λ^2^ in Equation (14) can be obtained from the following formula:

(15) $$\begin{array}{l} {\hat{\lambda }}^{2}\text{=}{\hat{\gamma }}_{0}\text{+}2\overset{q}{\underset{i=1}{\sum }}k\left(\frac{i}{q}\right){\hat{\gamma }}_{i} \end{array}$$

where γ_*i*_ is the *i* autocovariance function of ε_*t*_, and *k*(▪) is the Bartlett kernel function. *q* is the bandwidth parameter. According to the method of Newey and West (35), we take *q* = 4 × (*n*/100)^2/9^ as the initial value and select the bandwidth automatically decided by data. Mayoral (2) proved that $R_{b}^{f}\left({\hat{d}}_{n}\right)$ is a uniform statistics.

For the initial estimate of fractionally integrated order of a single sequence and the test under the null hypothesis that $\hat{d}=1$, the Monte Carlo and Bootstrap critical values of the statistics $R_{b}^{f}\left({\hat{d}}_{T}\right)$ are given by means of 20,000 repetitions of the simulation of the time series data with sample size of 57. If the value of the statistic $R_{b}^{f}\left({\hat{d}}_{n}\right)$ is less than a critical value at a confidence level, this is evidence to reject the null hypothesis, which means that the time series is pseudo long-memory.

### Identification of Time Point of Structural Mutation of Convergence Path of Relative Environmental Indicators per Capita

Although the test method proposed by Mayoral (2) has good test effect in identifying true long- memory (including infinite-memory, i.e., unit root processes) processes from pseudo long-memory ones, it is not a accurate break point detection method (23). This means that it can only identify whether a process is a long-term memory (non-stationary process) or a stationary process with a mean shift, but cannot identify the exact location of the break point in the case of “pseudo long-memory processes.” Therefore, it is necessary to identify the specific positions of all break points in the sequence by perfecting the above-mentioned test method. For this purpose, this study uses the null hypothesis *H*_0_ proposed by Berkes et al. (1), shown by the Equation (16):

(16) $$\begin{array}{l} \{\begin{array}{c} y_{t}=\mu +x_{t},1<t<k^{*}\\ y_{t}=\mu +\Delta +x_{t}\;k^{*}+1<t<n \end{array} \end{array}$$

where k^*^ is a point in time when the mean-shift happens, and μ and μ + Δ is the unknown mean and shift of the potential data generated process respectively. The sequence {*x*_*t*_} is a fourth-order stationary process with a mean of zero. This study uses the non-parametric CUSUM statistics proposed by Berkes et al. (1) and Aue et al. (36) to test *H*_0_ listed below.

*H*_0_: The observations *y*_1_, *y*_2_, ···, *y*_*n*_ follow the mean-shift process (21) and *x*_*t*_ is the stationary process with a weak auto-correlated structure.

*H*_1_: The observations *y*_1_, *y*_2_, ···, *y*_*n*_ are a long memory process, i.e. their potential model setting satisfies Equation (12).

For defining hypothesis test statistics, we apply the main ideas of Berkes et al. (1) to introduce a break point that occurs at time point ${\hat{k}}^{*}$, and estimate it by the following equation:

(17) $$\begin{array}{l} {\hat{k}}^{*}=\\ \min\left\{k:\underset{1<i<n}{\max}|\underset{1\leq j\leq n}{\sum }y_{j}-\frac{i}{n}\underset{1\leq j\leq n}{\sum }y_{j}|=|\underset{1\leq j\leq k}{\sum }y_{j}-\frac{k}{n}\underset{1\leq j\leq n}{\sum }y_{j}|\right\} \end{array}$$

Since the identification program is designed for a single break point, we take the minimum {▪} in the algorithm for detecting possible breakpoints to obtain the position of the strongest mean shift point. Therefore, the entire sample is divided into two sub-samples dependent on the break point ${\hat{k}}^{*}$. In each sub-sample, the *T*_*n*_ statistic can be constructed according to the following two formulas:

(18) $$\begin{array}{l} T_{n,1}=\frac{1}{s_{n,1}}{{\hat{k}}^{*}}^{-\frac{1}{2}}\underset{1\leq k\leq {\hat{k}}^{*}}{\max}|\underset{1\leq i\leq k}{\sum }y_{i}-\frac{k}{{\hat{k}}^{*}}\underset{1\leq i\leq n}{\sum }y_{i}| \end{array}$$

(19) $$\begin{array}{l} T_{n,2}=\frac{1}{s_{n,2}}{\left(n-{\hat{k}}^{*}\right)}^{-\frac{1}{2}}\underset{{\hat{k}}^{*}<k\leq n}{\max}|\underset{{\hat{k}}^{*}<i\leq k}{\sum }y_{i}-\frac{k-{\hat{k}}^{*}}{n-{\hat{k}}^{*}}\underset{{\hat{k}}^{*}<i\leq n}{\sum }y_{i}| \end{array}$$

In the above equations, the CUSUM statistics is standardized by long-term standard deviations *s*_*n*,1_ and *s*_*n*,2_ respectively. *s*_*n*,1_ is the standard deviation calculated based on the first piece of sample data $y_{1},y_{2},\cdot \cdot \cdot ,y_{\hat{k}}$; *s*_*n*,2_ is the standard deviation calculated based on the second part of sample data $y_{\hat{k}+1},y_{\hat{k}+2},\cdot \cdot \cdot ,y_{n}$. There are several different methods to estimate the long-term variance $s_{n}^{2}$. For example, if the series {*y*_*t*_} has a serial correlation structure, it can be estimated according to $s_{n}^{2}$ as follows:

(20) $$\begin{array}{l} s_{n}^{2}={\hat{\gamma }}_{0}+2\underset{1\leq j\leq q\left(n\right)}{\sum }\omega_{j}\left(q\left(n\right)\right){\hat{\gamma }}_{j} \end{array}$$

The ${\hat{\gamma }}_{i}$ can be calculated as follows:

(21) $$\begin{array}{l} \frac{1}{n}\underset{1\leq i\leq n-j}{\sum }\left(y_{t}-{ȳ}_{n}\right)\left(y_{t+j}-{ȳ}_{n}\right) \end{array}$$

Where ω_*j*_(▪) is a weighting kernel function, and *q*(*n*) is the bandwidth of the kernel function ω_*j*_(▪). Andrews (34) discussed seven different types of kernel functions that can be used to calculate the HAC estimator. We select the “Bartlett” kernel function ω_*j*_(*q*) = 1 − *j*/(*q* + 1) according to Berkes et al. (1) and Aue et al. (36) with the optimal bandwidth used by Newey West in the selected kernel function. Once the *T*_*n*_ statistics of the two sub-samples are obtained, the following *M*_*n*_ statistics can be constructed:

(22) $$\begin{array}{l} M_{n}=\max\left\{T_{n,1},T_{n,2}\right\} \end{array}$$

*T*_*n*_ can be used to discriminate whether the sample has been generated by a short or long-range dependent stationary process. Hence, if we split the sample at time ${\hat{k}}^{*}$, which is close to the true change-point k^*^ asymptotically we can assume that $y_{1},y_{2},\cdots \;,y_{k^{*}}$ and $y_{k^{*}+1},y_{k^{*}+2},\cdots \;,y_{n}$ are samples from a stationary sequence with a constant mean. Thus, we can use *T*_*n*,1_ and *T*_*n*,2_ to test if the samples $y_{1},y_{2},\cdots \;,y_{k^{*}}$ and $y_{k^{*}+1},y_{k^{*}+2},\cdots \;,y_{n}$ have been generated by a short-range or long-range dependent process. The CUSUM statistic compares the sample mean of the first k^*^ observations with the sample mean of the last (*n* − *k*) observations. If we assume a change in the mean at time k^*^, the absolute value of the difference of the means should be large for *k* = k^*^.

Berkes et al. (1) derived the following asymptotic distributions of the statistics *M*_*n*_ under the null hypothesis:

(23) $$\begin{array}{l} M_{n}\to^{d}\max\left\{\underset{0\leq t\leq 1}{\sup}|B^{\left(1\right)}\left(t\right)|,\underset{0\leq t\leq 1}{\sup}|B^{\left(2\right)}\left(t\right)|\right\} \end{array}$$

where *B*^(1)^(*t*) and *B*^(2)^(*t*) is a Brown bridge corresponding to two sub-samples, respectively. If the relative environmental indicator per capita is weakly dependent (there is no change in mean), the statistics *M*_*n*_ will converge to the upper bound of the Brown bridge. However, if an environmental indicator fellows a mean shift or a long memory process, *M*_*n*_ → ∞. The latter case is referred to as a false rejection of the null hypothesis that there is no change in mean. Because the dataset used here is not large enough to achieve an approximately asymptotic distribution. Therefore, we calculate the asymptotically critical values under *H*_0_ at the significance level of 10, 5 and 1% respectively. If *M*_*n*_ exceeds the critical value at a significance level, the null hypothesis cannot be rejected, and the breakpoint identified at the corresponding significance level is reasonable.

**Figure 1:**
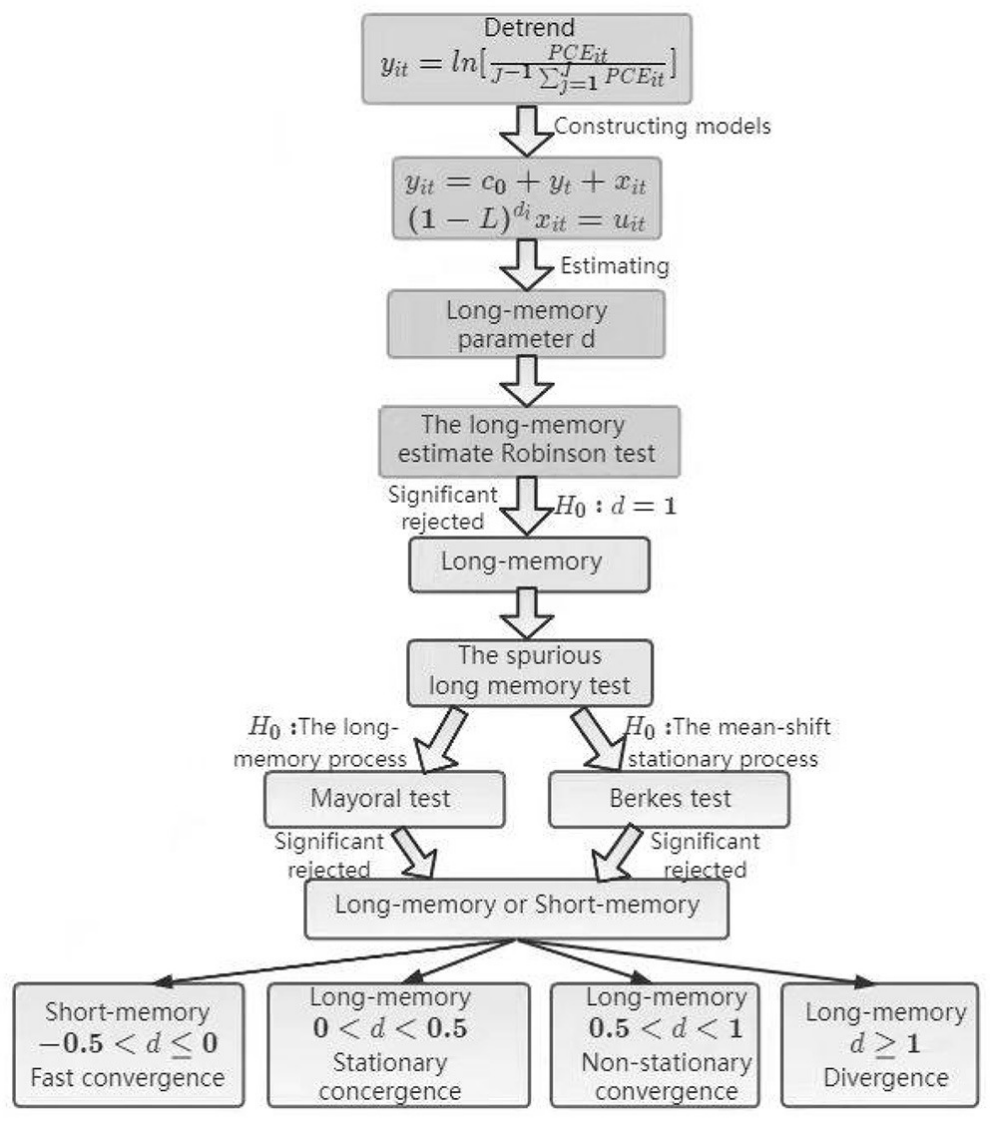
A flow diagram for testing the long-memory of ecological indicators.

This paper follows the procedure shown in Figure 1 to test whether ecological indicators are convergent in BRICS countries.

## Data

**Figure 2:**
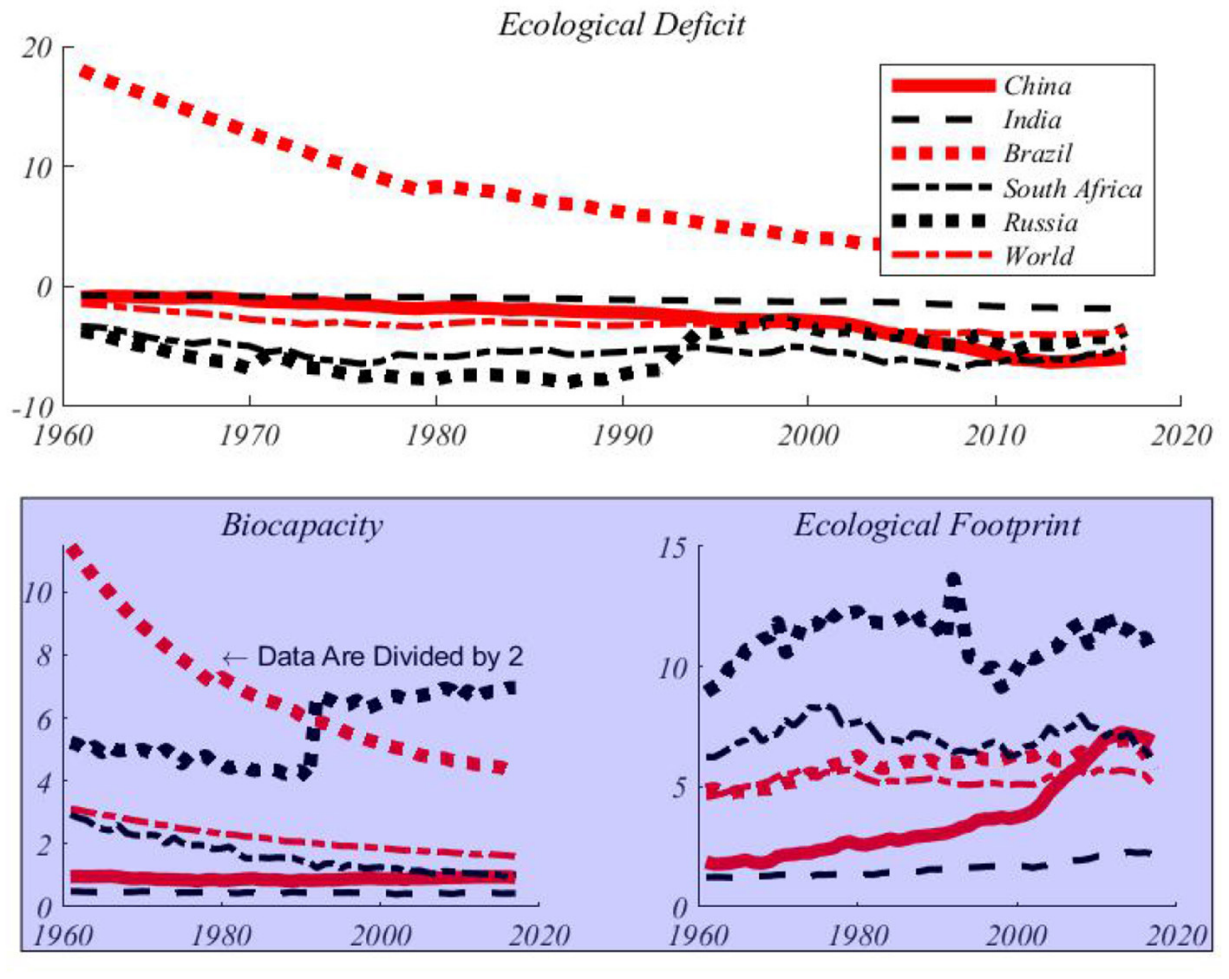
Per capita environmental indicators for BRICS and the world (gha). Russian data before 1989 are replaced with data from the former Soviet Union. So you can see from the fifth chart that there is a jump in 1989.

The data used in this paper are from National Footprint Accounts (NFAs). NFAs provides global and national annual accounting of biocapacity (BC) and ecological footprint (EF). This paper collects annual data of EF and BC per capita at the national level. The data range is from 1961 to 2017, with a total time span of 57 years. Figure 2 shows the trend of per capita ecological footprints and ecological carrying capacities of the BRICS countries (China, Brazil, India, Russia and South Africa) and the world. During the sample period, EFs are larger than the quantities enough to ensure sustainable development in BRICS and the world. In other words, the existing productive land and other resources are insufficient to meet the needs of human food, housing and CO_2_ absorption. During this period, around the worldwide, per capita EF increases by nearly 120%. BC per capita decreases by 50% during the same period. In 2017 the ecological deficit fell to 5.26 gha (a measure unit of ecological footprint and ecological carrying capacity), while EF almost doubled. This shows that human activities have been imposing great pressures on the nature. Although the ecological footprint can maintain the trend of not decreasing, the ecological carrying capacity decreases rapidly and the ecological deficit continues to increase every year. All three environmental indicators reveal that globally, human beings consume more resources with ecological productivity than available. For example, from 1961 to 2017, the ecological carrying capacity per capita decreases by 41.4%, from 3.12 gha to 1.83 gha per capita. This fact means that resources available per capita have been decreasing and more people are competing for limited resources (37).

Figure 1 shows that the world's per capita EF was stable in the 1980s and 1990s, and has shown an upward trend since the beginning of the twenty-first century, while per capita BC decreased gradually. Since 1961, the world EF per capita has been steadily increasing at a rate of 2.1% per year, almost tripling from 4.62 gha to 5.24 gha in 2017. Figure 2 also shows the trend of the per capita EFs and ecological carrying capacities of BRICS countries over time. In most countries, the per capita EFs continue to increase, while the per capita BCs are gradually decreasing or remain unchanged. With the rapid economic development of the BRICS countries, the problem of environmental pollution is becoming more and more serious. In 2017, all BRICS countries except Brazil were in ecological deficit. Compared with previous years, this deficit were very high in most of these countries. Therefore, it is necessary to study the stochastic evolution path of ecological indicators in BRICS. The EFs increase significantly in BRICS except for South Africa, especially after 1985. The per capita EFs have risen sharply in China and India, the biggest economies, indicating that these two countries are rapidly consuming natural resources. Although the trend of increase in per capita EF in Russia is not obvious, the level of per capita EF in Russia is higher than the sum of EFs in China and India. Although Brazil is the only BRICS country with an ecological surplus, its per capita BC is also declining at the fastest rate among BERICS, reaching the brink of an ecological deficit in 2017. Obviously, the level of economic development is closely related to the per capita EF. On the other hand, South Africa's ecological carrying capacity is obviously declining rapidly. In the past decade, China and South Africa have undergone higher ecological deficits than other countries. This means that the two countries with higher per capita incomes have put more pressures on the environment and faced a bigger problem of environmental deterioration. Environmental sustainability requires that the consumption of resources by the present generation cannot pose a threat to the needs of future generations. The imbalance between ecological footprints and ecological carrying capacities means that the present society is using the resources of future generations to live. In order to prevent the trend of further deterioration of the ecological deficit, the close cooperation between countries is required to take actions to improve the modes of production of enterprises and consumption patterns of human beings. Table 1 shows the descriptive statistics of BRICS and the world environmental indicators. From these summary statistics, we can see that there are also great differences in environmental indicators among BRICS. Russia has the highest per capita EF, while Brazil has the highest per capita BC.

**Table 1:** Summary statistics of per capita environmental indicators in BRICS and the world.

**Countries**		**China**	**India**	**Brazil**	**South Africa**	**Russia**	**World**
Mean	EF	3.635	1.588	5.889	7.097	11.153	5.306
	BC	0.896	0.448	13.358	1.638	5.596	2.182
Median	EF	2.974	1.557	6.033	7.065	11.241	5.268
	BC	0.892	0.449	12.50	1.544	5.100	2.074
Maximum	EF	7.273	2.289	6.931	8.394	13.671	5.734
	BC	0.973	0.492	22.892	2.935	6.963	3.116
Minimum	EF	1.779	1.199	4.707	6.095	8.037	4.564
	BC	0.819	0.394	8.609	0.958	4.083	1.598
Std. Dev.	EF	1.740	0.322	0.603	0.564	0.987	0.286
	BC	0.044	0.021	3.993	0.553	1.033	0.440
Skewness	EF	0.959	0.792	−0.515	0.417	−0.319	−0.440
	BC	0.183	0.005	0.773	0.702	0.068	0.568
Kurtosis	EF	2.598	2.517	2.423	2.601	2.780	2.730
	BC	1.914	2.913	2.547	2.311	1.251	2.141
JB	EF	9.128	6.516	3.305	2.029	1.082	2.009
	BC	3.118	0.018	6.158	5.805	7.312	4.812
P Value	EF	0.019	0.033	0.099	0.228	0.492	0.232
	BC	0.109	0.500	0.037	0.040	0.027	0.054

*(1) The last three lines represent the P values corresponding to the JB statistics. (2) “—” represent missing data*.

From Figure 2, it is also clear that the CFs and BCs are persistent, BCs downwards and CFs upwards. With respect to most of series, the processes might be not mean-reverting, and exhibit long memory properties presumably from the argument proposed by Granger and Joyeux (38) that the aggregation of first order Markov processes leads to a long memory process.

## Empirical Analysis

### Analysis of Convergence and Divergence of per Capita Ecological Indicators (EF and BC)

#### Convergence and Divergence of per Capita Ecological Indicators

Tables 2, 3 report the memory parameter estimates of the fractionally integrated processes estimated using LW, ELW and the ELW methods with detrended (or with removed breakpoints) for each environmental indicator sequence. The integration order of each environmental indicator in all countries is greater than 0, indicating that all sequences exhibit long memory characteristics. For each estimated memory parameter, a corresponding 95% confidence interval is provided. According to the confidence interval range of fractionally integrated proesses' memory parameters, it can be found that, in general, the confidence intervals of the memory parameter estimates of per capita ecological footprints in all countries have low bounds higher than 0.5, indicating that these indicators may be non-stationary. However, the low bounds of the confidence intervals of the per capita BC memory parameter estimates of BRICS are less than 0.5, which means that the per capita BCs may be stationary. For most countries, the memory parameter estimates of the ecological footprints per capita are greater than 1, which means that they will not converge to their common average of BRICS. Therefore, it is possible to reject both pure stationarity and unit root. In some cases, the effect of a given random shock will be temporary, but the sequence tends to return to its trend and converges slowly at a slower rate than a pure stationary sequence. The memory parameter of per capita BC sequences in BRICS are <1, indicating that these sequences are mean reverting (i.e., convergence). These insights can be analyzed in detail as follows.

**Table 2:** Long memory parameter estimates of per capita EFs in BRICS.

	**LW**	**ELW**	**2ELWd**	**De-breakpointed 2ELWd**
China	0.980	1.205	1.190	1.116
	[0.973 1.172]	[0.761 1.412]	[0.799 1.359]	[0.696 1.302]
$H_{0}:\hat{d}=1$	−26.567	−48.058	−47.157	−41.832
MC C V	17.959(+)	13.787(+)	24.458(+)	16.041(+)
Bootstrap CV	4.836(+)	4.809(+)	4.877(+)	4.185(+)
India	0.889	1.184	1.182	1.002
	[0.726 1.157]	[0.792 1.351]	[0.772 1.357]	[0.582 1.167]
$H_{0}:\hat{d}=1$	58.948	4.140	−23.313	15.711
MC CV	8.495(+)	11.109(+)	12.052(+)	15.041(+)
Bootstrap CV	3.656(+)	3.205(+)	0.364(+)	3.661(–)
Brazil	0.762	1.148	1.143	0.932
	[0.579 1.068]	[0.766 1.300]	[0.743 1.336]	[0.480 1.114]
$H_{0}:\hat{d}=1$	−0.856	−1.777	−0.858	−1.238
MC CV	11.436(+)	12.304(+)	9.135(+)	13.402(+)
Bootstrap CV	3.957(+)	3.973(+)	3.966(+)	3.926(+)
South Africa	0.880	0.934	0.892	0.853
	[0.707 1.162]	[0.524 1.14]	[0.459 1.062]	[0.853 1.009]
$H_{0}:\hat{d}=1$	3.277	−1.893	2.943	4.072
MC CV	13.574(+)	12.041(–)	11.442(–)	11.391(–)
Bootstrap CV	3.078(+)	3.049(–)	3.076(–)	3.067(–)
Russia	0.823	0.924	0.869	0.643
	[0.632 1.125]	[0.561 1.067]	[0.477 1.017]	[0.202 0.737]
$H_{0}:\hat{d}=1$	53.153	28.168	40.206	155.513
MC CV	8.115(–)	13.451(–)	11.634(–)	13.237(–)
Bootstrap CV	3.335(–)	3.351(–)	3.343(–)	3.253(–)

*In this paper, Bootstrap percentile -t confidence intervals are used for evaluating the estimates of memory parameters, at 95% confidence level. (+) represents rejecting null hypothesis; while (–) represents accepting null hypothesis. We make inference dependent on 5% significant level. With respect to the Robinson statistics represented by Equation (4), empirical critical values are generated by 2000 Monte Carlo repetitions and 2000 Bootstrap resamples, respectively, the corresponding contains of following tables are the same as this table*.

**Table 3:** Long memory parameter estimates of per capita BCs in BRICS.

	**LW**	**ELW**	**2ELWd**	**De-breakpointed 2ELWd**
China	0.889	0.752	0.844	0.663
	[0.775 1.008]	[0.369 0.752]	[0.395 0.998]	[0.192 0.787]
$H_{0}:\hat{d}=1$	231.241	608.571	326.933	1068.545
MC CV	18.627 (–)	14.856 (–)	14.382 (–)	20.292 (–)
Bootstrap CV	3.757 (–)	3.835 (–)	3.801 (–)	3.776 (–)
India	0.824	0.811	0.874	0.668
	[0.618 0.987]	[0.352 0.895]	[0.461 1.041]	[0.257 0.773]
$H_{0}:\hat{d}=1$	366.628	396.608	266.935	940.048
MC CV	22.274 (–)	9.843 (–)	11.593 (–)	11.695 (–)
Bootstrap CV	4.080 (–)	4.905 (–)	4.051 (–)	4.056 (–)
Brazil	0.903	1.047	1.017	0.290
	[0.835 1.067]	[0.646 1.173]	[0.618 1.110]	[-0.236 0.383]
$H_{0}:\hat{d}=1$	152.153	−112.29	−68.358	155.375
MC CV	9.396 (–)	17.748(+)	13.938(+)	11.759 (–)
Bootstrap CV	3.907 (–)	3.956(+)	3.965(+)	3.946 (–)
South Africa	0.824	0.733	0.586	0.018
	[0.605 1.046]	[0.351 0.745]	[0.172 0.696]	[-0.375 0.196]
$H_{0}:\hat{d}=1$	48.674	97.146	290.986	169.625
MC CV	14.134 (–)	13.445 (–)	19.715 (–)	8.285 (–)
Bootstrap CV	2.919 (–)	2.959 (–)	2.933 (–)	2.958 (–)
Russia	0.907	0.948	0.928	0.287
	[0.819 1.113]	[0.534 1.064]	[0.380 1.024]	[-0.273 0.421]
$H_{0}:\hat{d}=1$	3.642	1.860	2.667	319.372
MC CV	10.101(+)	9.221(+)	10.610(+)	9.919(+)
Bootstrap CV	3.512 (–)	3.494(+)	3.459(+)	3.514(+)

As for testing per capita ecological indicators' convergence, the Monte Carlo critical values and bootstrap critical values of the Robinson test make the consistent conclusion, indicating that the per capita ecological footprint sequences of China and India are divergent; The per capita ecological footprints of Russia and South Africa are convergent, but the processes of convergence are quite slow; The per capita ecological footprint of Brazil shows a very weak convergence dynamics. All countries expect for South Africa have memory parameters of per capita carbon footprint series significantly >1, which means that the per capita carbon footprints of these four countries are far from convergence. China's relative ecological footprint per capita has the highest persistence ($\hat{d}=1.190$); Russia's per capita relative ecological footprint has the lowest persistence ($\hat{d}=0.643$), indicating that Russia's per capita carbon dioxide emissions are serious. The confidence interval for the estimated fractionally integrated process parameter (long-memory parameter) is very narrow and generally located within a positive interval range. For the relative ecological footprints per capita, the upper bound of the confidence intervals are generally >0.5, indicating that the ecological footprints are non-stationary and some are even mean-reverting, but exhibit long memory characteristics. The two environmental indicators in other countries do not have the feature of mean reversion and are not convergent. For South Africa and Russia, the inference results, i.e., convergence or divergence, are very consistent, no matter Monte Carlo or bootstrap approach is used to statistically infer. Specifically, the range of estimates $\hat{d}$ is between 0.49 and 0.72 (statistically always different from 1), indicating long memory and mean- reverting (convergent) processes, but not covariance stationary processes. Therefore, the per capita relative ecological footprints of China, India and Brazil are not convergent, while those indicators of South Africa and Russia are convergent, although the convergence will take quite long time ($\hat{d}=1.190$).

However, as for the ecological carrying capacities, it is another story. The upper bounds of the memory parameters $\hat{d}$ of the BC sequences per capita in most of BRICS countries are greater than 0.5, but less than 1, which indicates that these sequences are mean-reverting, but of not stationary. These results show that in all BRICS countries the BC sequences are convergent, with quite different convergent rates. In China and India, the BC sequences have the longest memory degree, and the estimated memory parameters are $\hat{d}=0.668$ and $\hat{d}=0.663$, respectively, which means the two countries have the slowest convergent paths. However, the memory parameter of South African per capita BC sequence is that $\hat{d}=0.018$, in other words no long memory characteristic, i.e. converging quickly. The memory parameter estimate of the BC sequence of Russia is less than 0.5 ($\hat{d}=0.287$). The analysis above means that the per capita BC is a stationary process, but a long memory process, converging in a relatively fast rate. From the policy perspective, as long as the government adopts environmental protection and resource use policies designed appropriately, the decline trends of the per capita ecological carrying capacities of BRICS can be reversed and eventually converge. It is not as stubborn as the worsening trend of the ecological footprints, which have put tremendous pressures on the governments of BRICS.

#### Statistical Test of Convergence and Divergence of per Capita Ecological Indicators (EF and BC)

**Figure 3:**
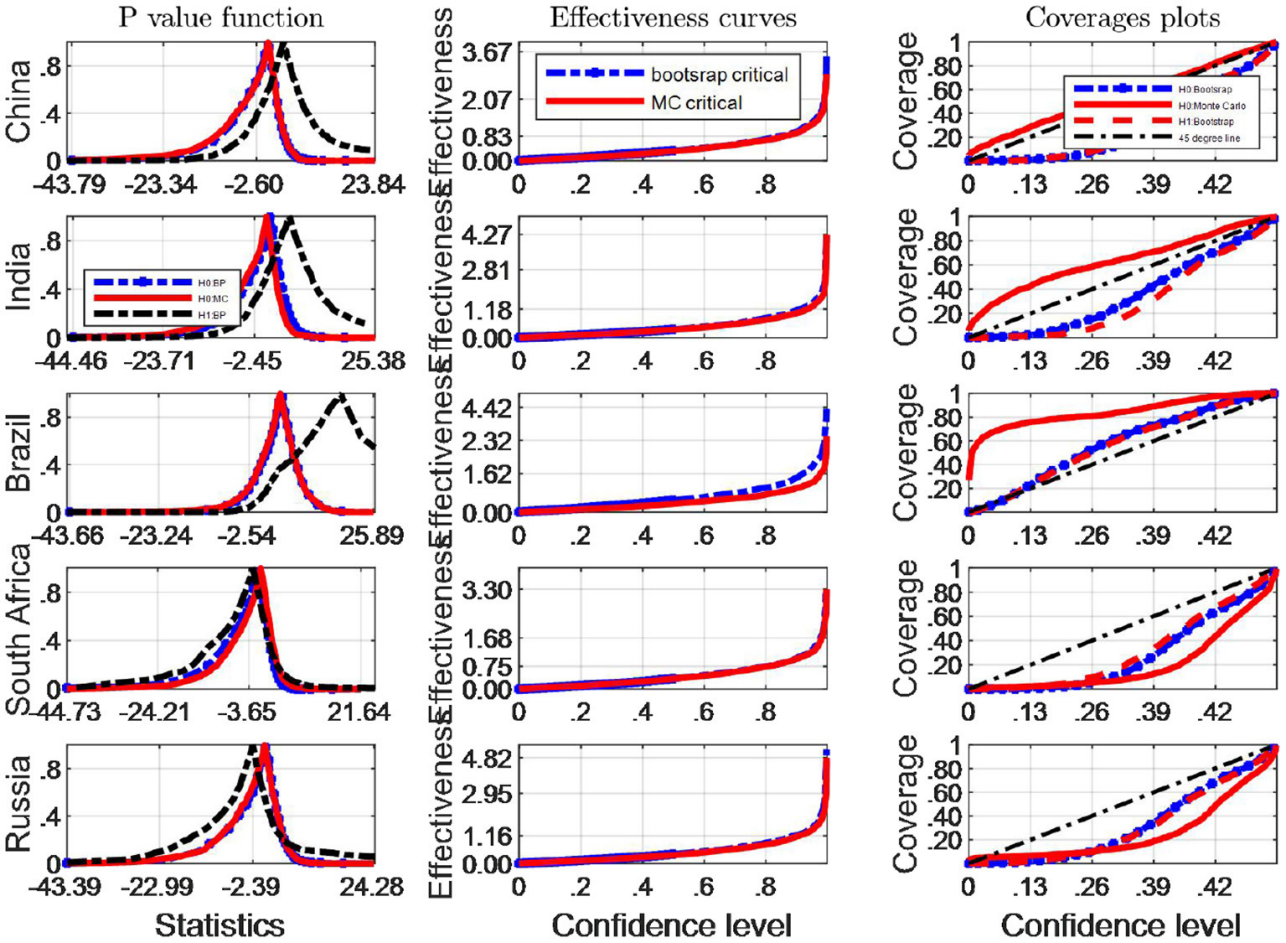
A comparison of confidence intervals of Monte Carlo and counterparts of bootstrap for the Robinson test using EF indicators.

**Figure 4:**
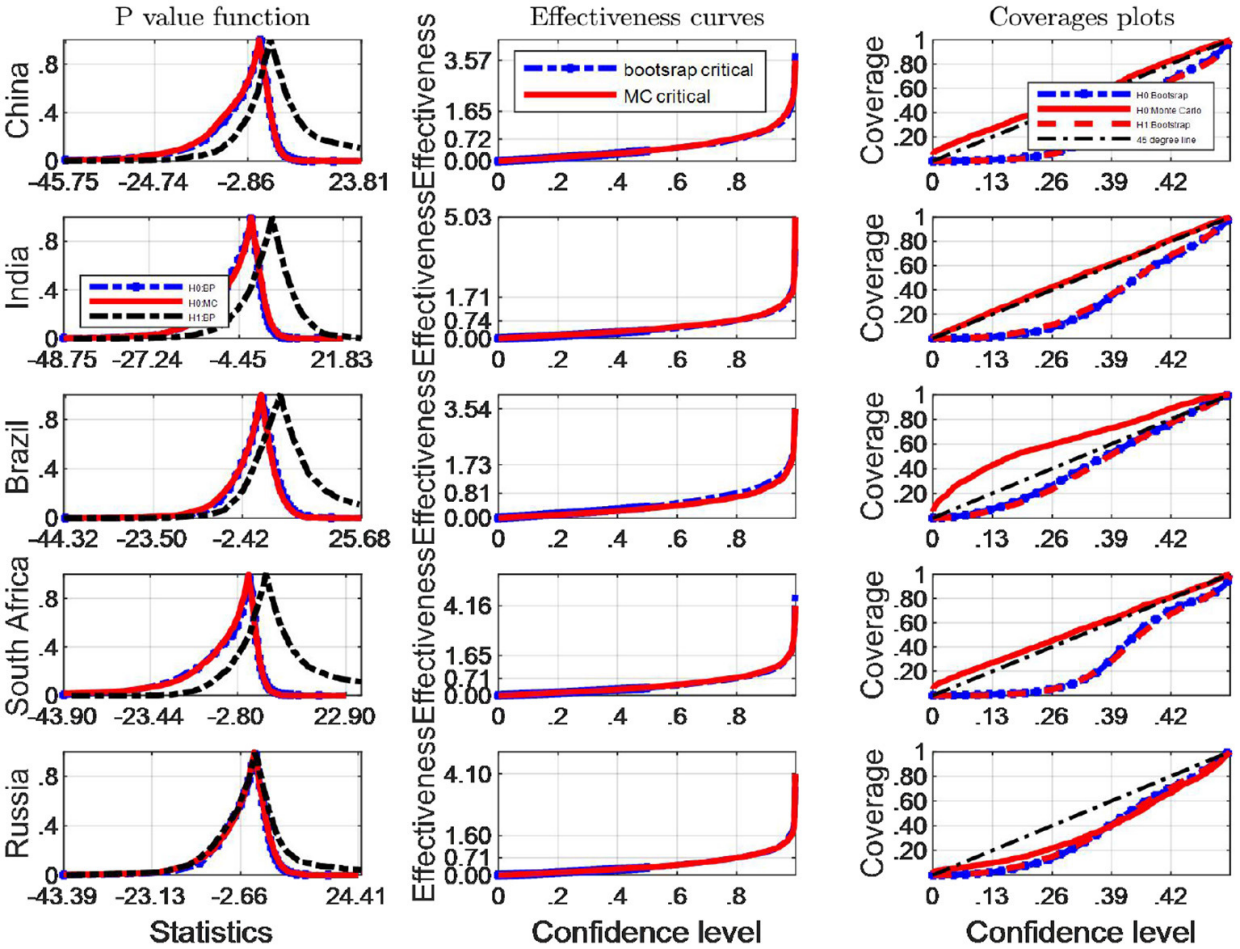
A comparison of confidence intervals of Monte Carlo and counterparts of bootstrap for the Robinson test using BC indicators.

We now turn our attention to investigating the finite sample simultaneous coverage probability and effectiveness of the critical intervals for the Robinson (33)'s tests. This kind of graphic is very useful for choosing among critical values that have reasonable coverage distortions and effectiveness improvements: it permits to make arbitrage between the coverage distortion and the true effectiveness for each critical values and then to chose the most appropriate. More specifically, we do both the IID and Monte Carlo and bootstrap sampling respectively for 2,000 independent replications with *n* = 64. In each replication, we calculate uncorrected marginal intervals. We then plot the point estimate of the coverage and effectiveness along with simultaneous confidence regions in Figures 3, 4.

We consider coverage plots as suggested by de Peretti and Siani (5) defined as follows. For a nominal coverage x the coverage is calculated as *x*_*s*_ = 1 − 2 min{*pv*, 1 − *pv*}, where *pv* is equal to

(24) $$\begin{array}{l} pv=\frac{1}{B}\overset{B}{\underset{b=1}{\sum }}I\left(\hat{\tau }\leq \tau_{0}\right). \end{array}$$

where pv is the estimated probability distribution function at the true Robinson (27)'s statistics $\hat{\tau }$, using any of the bootstrap and Monte Carlo strategies described above. τ_0_ is the correspoding critical value, and *B* simulation repetitions. The closer the coverage line is to 45 degree line the better the strategy used to approximate the distribution of Robinson statistics performs. The third column of Figures 2, 4 presents the coverage plots of the interval based on inverting (single) bootstrap tests for the five BRICS countries. Not only is the frequency of coverages important to assess the performance of a CI but its length is also a relevant measure of its effectiveness [see (5)]. If two CIs have the same confidence level the shorter is preferable, indicating higher precision in the estimation. The second column of Figures 3, 4 presents the confidence level-effectiveness curves of the interval based on inverting (single) bootstrap tests based on the parameter estimates of the five counties. On the basis of confidence level-effectiveness curves, two critical values seem to have the same effectiveness (expect for Brazil) and to be dominated differently. Following the results on Figures 3, 4, the Bootstrap method has the most satisfactory “true” effectiveness for Brazil.

As is analyzed above, Figure 3 shows clearly that MC critical values should be resorted to judge the Robinson tests for EF indicators, and the confidence interval based on inverting bootstrap tests is by far the best method on the basis of coverage accuracy criterion in China and India. However, the bootstrap critical values should be used to infer Robinson tests in other BRICS countries since these coverage curves approaches 45 degree lines closer, and their confidence intervals are narrower.

It is obvious that the Bootstrap critical value does not perform well for judging the statistic hypothesis test of BC indicators in almost all BRICS countries (shown by Figure 4) since the MC plot is closer to the 45 degree line. Consequently, in these countries MC critical values should be employed to infer Robinson test. However, in Brazil, Bootstrap critical values are more appropriate tools to decide the inference of Robinson test at a significance level.

### Pseudo-Long Memory Test (Mayoral Test) for Per Capita Environmental Indicator Sequences

#### Pseudo-Long Memory Judgment of Per Capita Environmental Index Sequence

In the second stage, for achieving the more accurate estimates of the long memory or infinite memory parameters in BRICS, we control the deterministic trend and possible breakpoints of the ecological indicator sequences. Table 4 provides the statistical test and long-memory parameter estimation results. The Mayoral test shown by Table 4 identifies which hypothesis is true: non-stationary long memory vs. *I*(0) + breakpoints for the environmental indicator series. The purpose of this test is to determine the cause resulting in the observed long-term memory processes: whether it is the result of a highly persistent shock, or whether those rare and unexpected events have changed the sequence's potential dynamics, inducing structural breakpoints. For each country, the first line reports the memory parameter estimates, while the second and third lines report the critical values generated by the corresponding methods, i. e. Monte Carlo and Bootstrap approaches. We discuss presence or absence of structural breakpoints in the context of ARFIMA models, and are particularly interested in the effect of structural breakpoints on fractional integration parameter estimates. The results in Table 4 show that the inference of whether or not per capita environmental indicator sequences are long memory is very robust. According to the statistics $R_{b}^{f}\left({\hat{d}}_{T}\right)$ of per capita ecological footprints of BRICS, there is no evidence to reject the null hypothesis that the per capita EF sequences are fractionally integrated process (FI) with long memory characteristics in BRICS countries. Therefore, we can conclude safely that these fractionally integrated sequences might be non-covariance-stationary, with no significant breakpoints in the deterministic trend detected (as shown in Figure 1).

**Table 4:** Pseudo-long memory Mayoral test of relative per capita environmental indicators in BRICS.

	**LW**		**ELW**		**De-trented (breakpoint) 2ELWd**	
	**EF**	**BC**	**EF**	**BC**	**EF**	**BC**
**China**						
Mayoral statistics	0.069	0.081	0.0112	0.255	0.023	0.530
MC CV	0.051	0.072	0.0095	0.214	0.018	0.438
Bootstrap CV	0.039	0.073	0.009	0.201	0.016	0.393
**India**						
Mayoral statistics	0.165	0.142	0.150	0.158	0.068	0.510
MC CV	0.085	0.105	0.007	0.116	0.033	0.386
Bootstrap CV	0.073	0.116	0.010	0.128	0.034	0.368
**Brazil**						
Mayoral statistics	0.908	0.170	0.042	0.051	0.244	13.891
MC CV	0.260	0.066	0.012	0.020	0.065	9.158 (–)
Bootstrap CV	0.189	0.067	0.013	0.025	0.055	7.924 (–)
**South Africa**						
Mayoral statistics	0.106	0.095	0.067	0.210	0.133	64.882
MC CV	0.068	0.085	0.043	0.184	0.085	74.315(–)
Bootstrap CV	0.080	0.119 (–)	0.054	0.234 (–)	0.096	76.952 (–)
**Russia**						
Mayoral statistics	0.154	0.135	0.067	0.095	0.562	8.83
MC CV	0.115 (–)	0.057 (–)	0.051	0.040	0.524	9.500 (–)
Bootstrap CV	0.119 (–)	0.065 (–)	0.058	0.049	0.468	8.238

*With respect to Mayoral statistics shown by Equation (14), empirical critical values are obtained by 2,000 repetitions of Monte Carlo simulation and 2,000 repetitions of bootstrap resample respectively. the corresponding contains of following tables are the same as this table*.

In China and India, with respect to the per capita BC sequences, we cannot reject the null hypothesis that the sequences of Equation (7) are long memory processes by the Mayoral test, depending Monte Carlo and bootstrap critical values. As shown in Figure 2, the per capita BCs are really long memory processes. For Brazil and South Africa, the Mayoral statistics is calculated by using the memory parameter estimates obtained from the detrended 2ELWd method. Both Monte Carlo and bootstrap critical values show that there is a breakpoint in per capita BCs, indicating that per capita BCs converge faster. Although the memory parameters estimated by the LW method in Russia show the existence of structural breakpoints, for the LW method is a relatively ineffective method, we generally prefer to the estimation results of the improved LW method. Therefore, it can be concluded that there is no breakpoint for the per capita BC in Russia. The economic implication of this conclusion is very clear: the Mayoral test indicates that there is no breakpoint in most countries' environmental indicators, which means that environmental indicators are highly persistent processes.

Therefore, on the whole, the overwhelming majority of BRICS countries have not undergone structural changes in the per capita environmental indicators, and they are long memory processes. Implications arising from the results above that international multilateral agreements such as the Kyoto Protocol and the Paris Agreement, which restrict greenhouse gas emissions and promote sustainable development, have not played a substantive role.

#### Pseudo-Long Memory Statistical Test of per Capita Environmental Indicator Sequences

**Figure 5:**
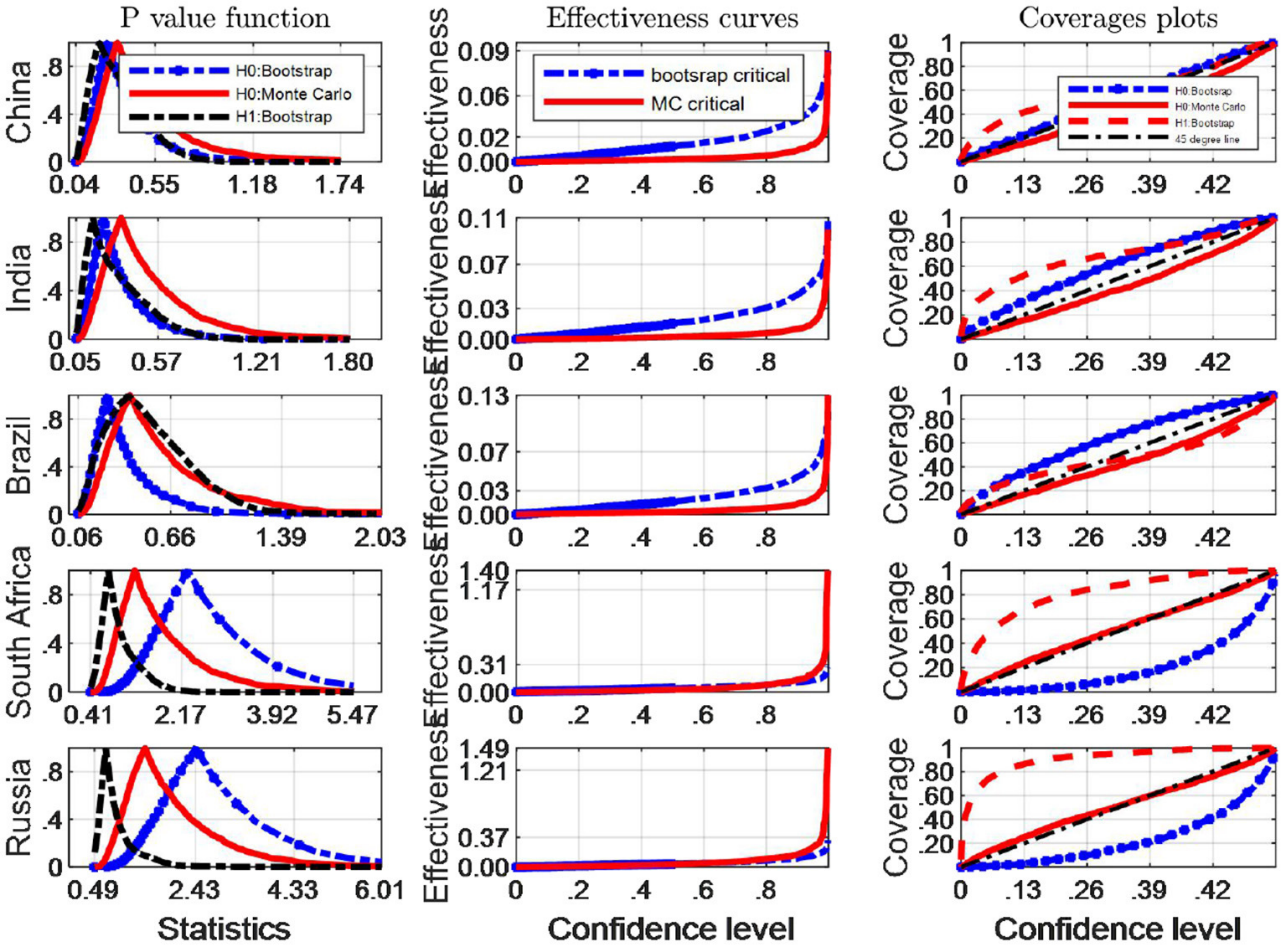
A comparison of confidence intervals of Monte Carlo and counterparts of bootstrap for the Mayoral test using EF indicators.

**Figure 6:**
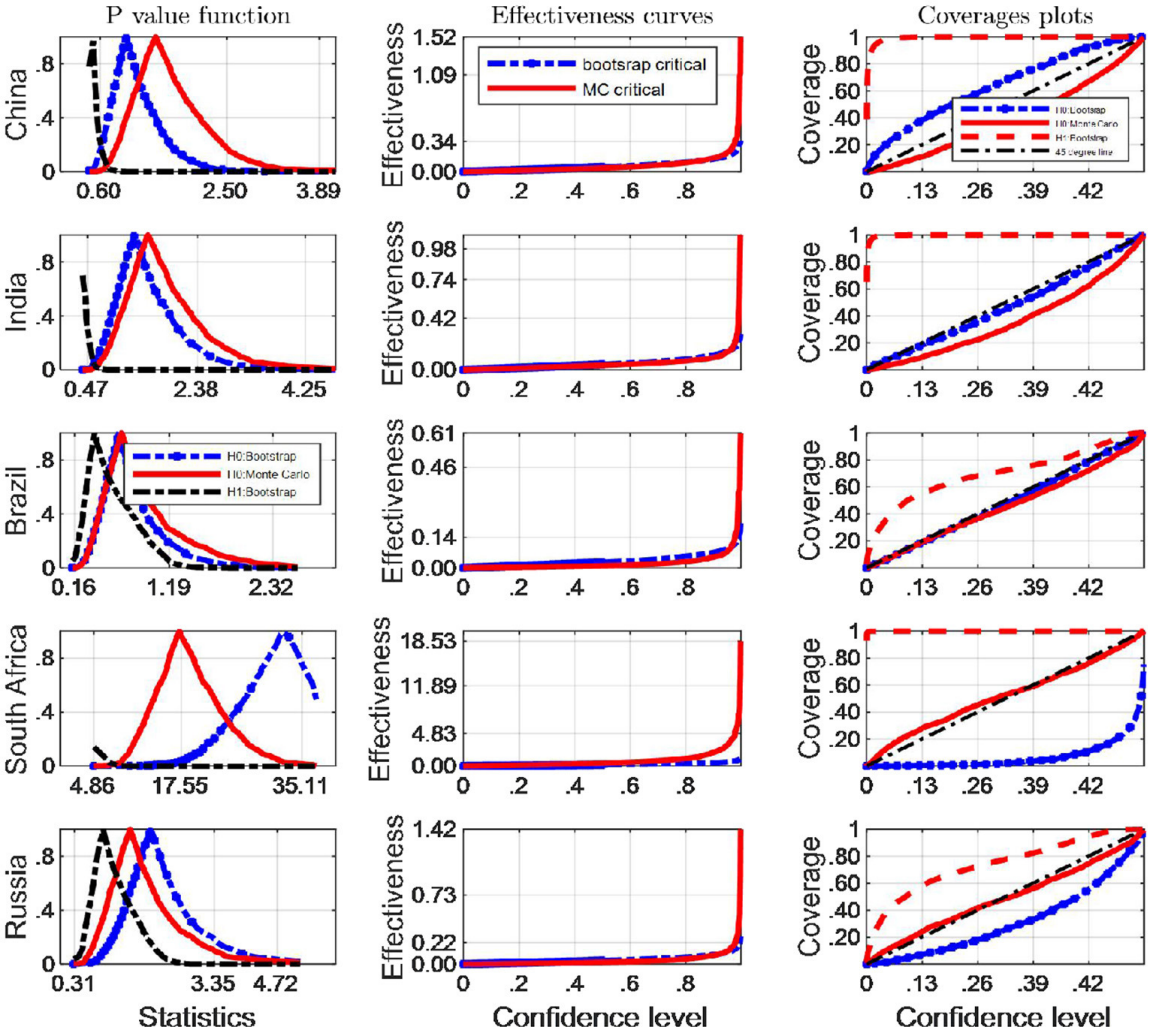
A comparison of confidence intervals of Monte Carlo and counterparts of bootstrap for the Mayoral test using BC indicators.

In this paper we use inverting (single) bootstrap tests to construct three confidence intervals named by H0: Monte Carlo, H0: Bootstrap and H1: Bootstrap (see notations shown by legends of Figures 5, 6) for the Mayoral test statistics in 5 BRICS countries and compare with each other and infer whether the H0 hypothesis hold at usual significant levels. The results presented in Figures 5, 6 suggest that CIs are constructed more profitably under H0 than under H1.

Of the three confidence intervals, the evaluation of H0: Monte Carlo and H0: Bootstrap is based on their coverage accuracy and average length. Column 2 and 3 of Figures 5 and 6 show average lengths and the coverage probabilities of the confidence intervals calculated for 2,000 runs, respectively. A shorter average length corresponds to a better effectiveness performance. Figure 5 shows that the Monte Carlo test statistics produces CIs that are longer (lower effectiveness) than CIs constructed using the bootstrap test statistics for EF indicator in China, India and Brazil; while MC critical values are of minor advantages over Bootstrap counterparts in South Africa and Russian based on effective curves.

The results set out in Figure 6 show that the confidence interval of the Mayoral test statistics built by Monte Carlo simulation has the better performance on coverage probability compared with that of the bootstrap method for Mayoral test in South Africa and Russia. Just the opposite is true to other BRICS countries. However, the results displayed in Figure 6 reveal that for the BC indicators' Mayoral test statistics, the average length of the Monte Carlo confidence intervals is longer than that produced by the Bootstrap method in Brazil, indicating worse effectiveness. The inference dependent of Bootstrap critical values is more effective in terms of higher coverage probability and shorter average length than Monte Carlo counterparts of other countries among BRICS. On the contrary, the average length of the Monte Carlo confidence intervals is shorter in South Africa, which means Monte Carlo critical values are more satisfactory inference tools. The effectiveness curves do not show the visually obvious difference in other three BRICS countries. Synthesizing the results of Figures 5, 6, and balancing the performance of the effectiveness and coverage probability, Monte Carlo critical values are appropriately able to be used in Mayoral test in South Africa and Russia. Thus, the exact bootstrap method is not recommended for identifying potential break points in the two countries. By contrast, bootstrap critical values are the preferred inference tools to Mayoral test in remaining countries. No matter what environmental indicators are tested, these conclusions from Figures 5, 6 are the same.

### Breakpoint Identification of per Capita Environmental Indicator Sequences (Berkes Test)

#### Breakpoint Judgment of per Capita Environmental Indicator Sequences

**Figure 7:**
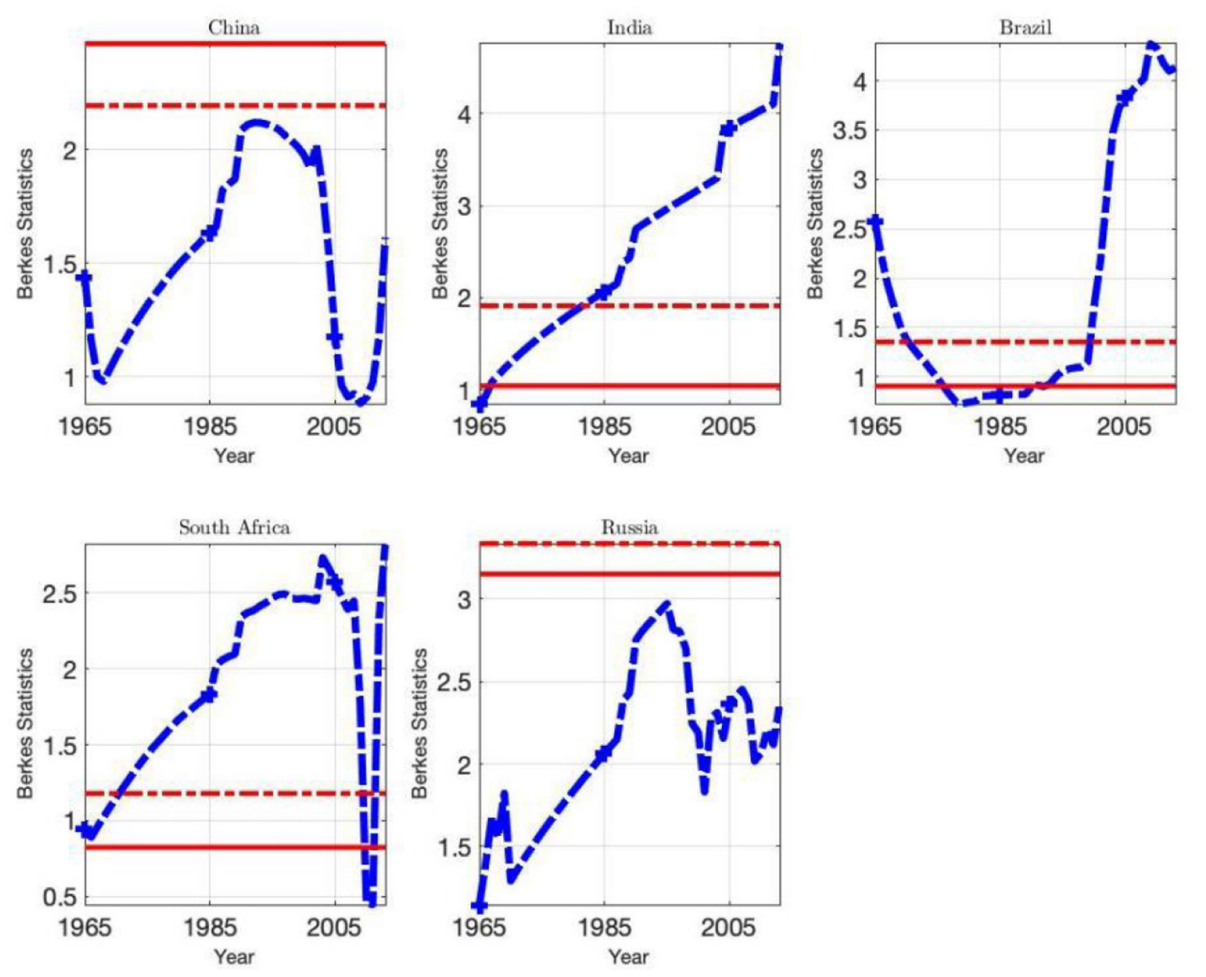
M_n_ statistics for BRICS. This graph plots Berkes statistics (blue lines) evolving over time. Red solid lines represent Monte Carlo critical values at 5% significant level; while red dash lines represent bootstrap critical values at 5% significant level. When red lines are located above the maxima of *M* statistics, Berkes test reject null hypothesis. With respect to Berkes statistics represented by Equation (22), empirical critical values are obtained by 2,000 repetitions of Monte Carlo simulation and 2,000 repetitions Bootstrap resample respectively.

In order to check the reliability of the results, this paper supplements the Mayoral (2) test by using the CUSUM statistic of Berkes et al. (1) to perform a further test so as to infer the existence of breakpoints and determine the locations of breakpoints. Table 5 shows the statistics *M*_*n*_ of Berkes et al. (1). Due to the good performance of small samples, we use the Bartlett kernel function and select the optimal bandwidth suggested by Newey-West (see Section **Convergence Pseudo-long Memory Test of Relative Environmental Indicators Per Capita**). The null hypothesis represented by Equation (10) indicates that there is no structural change in the deterministic trend of the ecological indicator sequence. Figure 7 shows how the ecological footprint statistics *M*_*n*_ for each country evolves over time. Due to the space limitation, the statistics *M*_*n*_ of the BCs per capita in BRICS countries over the sample years is not listed in the present paper. The statistics in Figure 7 and Table 5 shows that the maximum value of the Berkes statistics of the per capita EF sequences and per capita BC sequences of the BRICS countries will not be lower than critical values of themselves, which can reject the null hypothesis and there are not any break points identified. Therefore, within the sample region of 57 years, the ecological footprints per capita and BC sequences per capita are either covariance non-stationary process of mean reverting or unit root process, i.e., there is long memory.

**Table 5:** Pseudo long memory Berkes test of relative per capita environmental indicators in BRICS.

	**LW**		**ELW**		**De-trented (breakpoint) 2ELWd**	
	**EF**	**BC**	**EF**	**BC**	**EF**	**BC**
**China**						
Berkes Statistics	4.712	3.212	4.713	3.612	2.13	3.812
MC CV	0.665	1.732	0.668	1.730	2.665	1.729
Bootstrap CV	1.165	1.868	1.166	1.864	2.161	1.867
**India**						
Berkes Statistics	4.105	4.104	4.105	4.108	4.105	4.108
MC CV	1.055	1.857	1.048	1.858	1.038	1.858
Bootstrap CV	1.891	1.925	1.873	1.924	1.913	1.924
**Brazil**						
Berkes Statistics	4.375	4.105	4.375	4.105	4.374	4.105
MC CV	0.905	2.243	0.903	2.242	0.902	2.240
Bootstrap CV	1.333	2.291	1.340	2.283	1.350	2.286
**South Africa**						
Berkes Statistics	2.735	4.102	2.735	4.102	2.735	2.105
MC CV	0.823	2.616	0.824	2.616	0.822	2.616
Bootstrap CV	1.177	2.561	1.172	2.562	1.179	2.562
**Russia**						
Berkes Statistics	4.117	4.111	4.117	4.111	2.917	2.214
MC CV	0.849	1.481	0.848	1.479	3.251 (–)	2.491 (–)
Bootstrap CV	1.493	1.667	1.517	1.685	3.132 (–)	2.678 (–)

#### Breakpoint Statistical Test of per Capita Environmental Indicator Sequences

**Figure 8:**
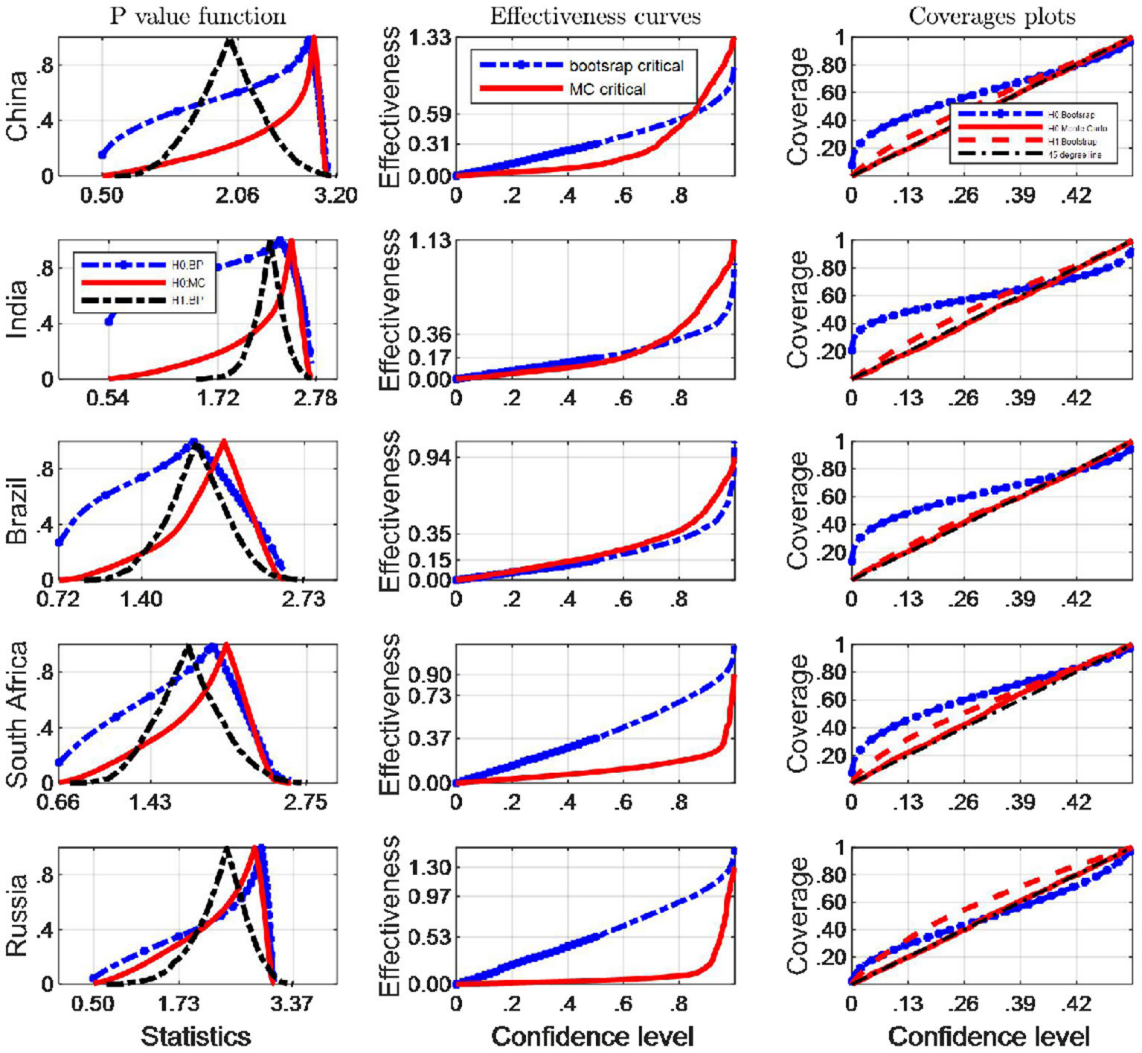
A comparison of confidence intervals of Monte Carlo and counterparts of bootstrap for the Berkes test using EF indicators.

**Figure 9:**
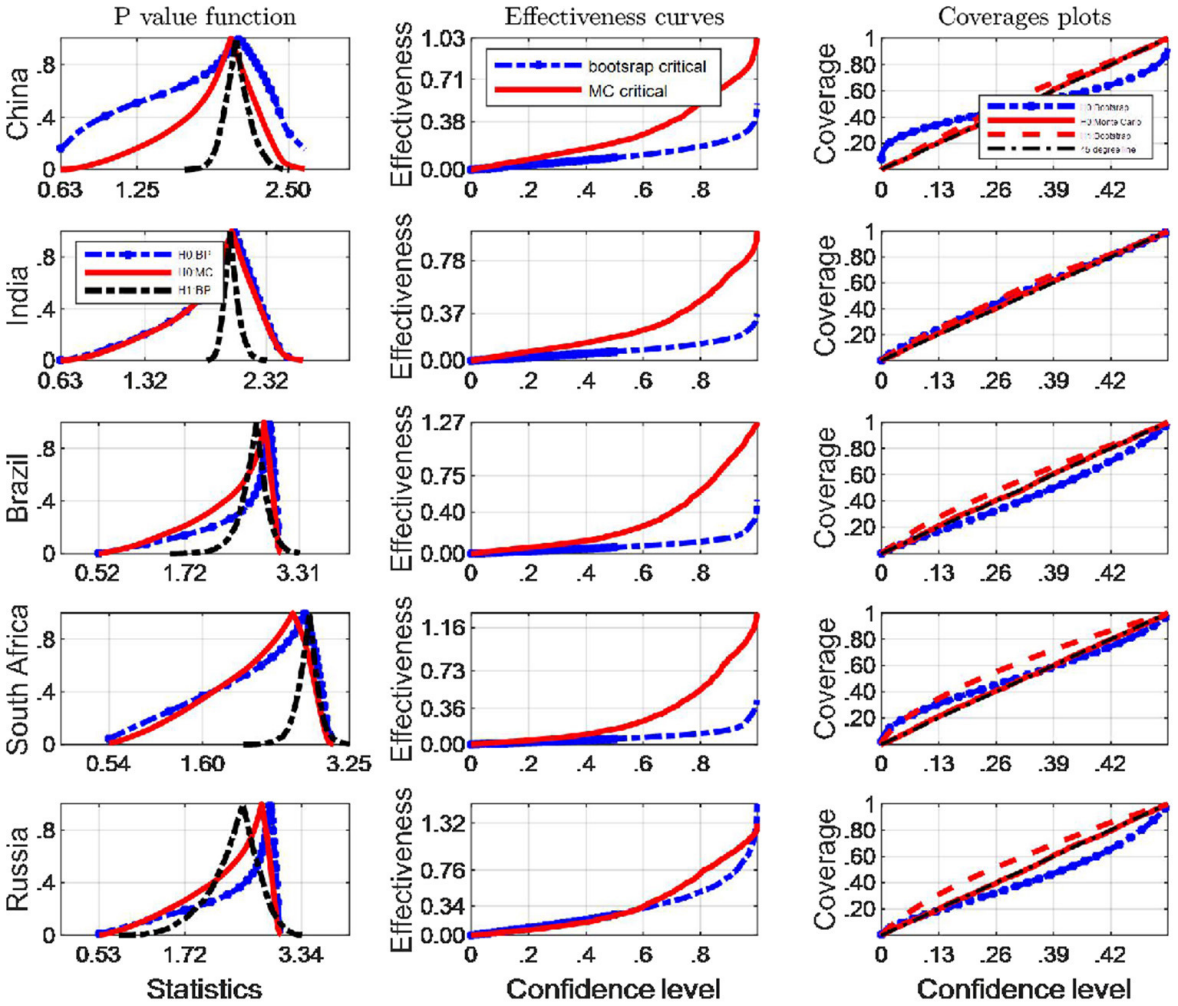
A comparison of confidence intervals of Monte Carlo and counterparts of bootstrap for the Berkes test using BC indicators.

Figures 8, 9 show coverage and effectiveness results for EF and BC indicators respectively, with separate plots for Monte Carlo and Bootstrap critical values. These two figures show that the Monte Carlo arbitrament methods tended to provide smaller confidence intervals with better coverage accuracy than the bootstrap arbitrament methods over almost all Berkes test statistics domains (judged by the first column and the third column of Figure 8).

The two figures again indicate that Monte Carlo method coverage probability generally compare favorably against those of Bootstrap method based on inverting Bootstrap CIs, and in particular Monte Carlo method intervals seem to emerge with slightly better coverage rates asymptotically from their evolving dynamics as well. However, the Monte Carlo method indiscernibly performs better than does the Bootstrap methods when the Berkes test methodology is employed to the Russian EF indicator and the South African BC indicator. These plots also indicate that, comparing with the Bootstrap method, although the Monte Carlo inference method has similar coverage rates over higher confidence levels, there are obvious systematic differences in overall interval lengths. Over lower confidence levels, we can identify similar effectiveness of the two inference methods generally.

When we test potential breakpoints occurring in BC indicator sequences, in terms of interval lengths, the Monte Carlo method has uniformly narrower intervals, especially when confidence levels are small (Figure 9). Following the results provided by Figure 8, the Monte Carlo critical values has the most satisfactory “true” effectiveness for the inference of potential break points in almost all the BRICS countries, expect for South Africa and Russia when EF indicators are tested. However, the difference between the average lengths of the various methods is not very large, especially over lower confidence levels. Consequently, the Monte Carlo critical values should be retained to judge whether a long-memory process is true or just a mean-shifted process, on the double basis of the coverage and the average length criteria. With respect to the BC indicator, the confidence intervals for critical values from the Monte Carlo methods are shorter than the bootstrap method at usual confidence levels, e.g., 95%.

Our simulation results indicate that when the BC indicators are tested if they are pseudo-long memory processes, the Monte Carlo critical values have the best performance based on both coverage probability compared with the bootstrap counterparts, since the coverage plots produced by MC critical values are closer to 45° lines. However, what the second column tells is that more statistory effectiveness curves are produced by bootstrap critical values expect for India and Brazil. A summary statement can be concluded that Monte Carlo CIs generally performed better than their bootstrap counterparts. Based on the analysis above, when the EF indicators are tested pseudo-long memory, the bootstrap critical values should be resorted to in South Africa and Russia, and the Monte Carlo critical values are appropriate to other BRICS countries. Therefore, this study recommends Monte Carlo confidence interval to quantify the uncertainty of the Berkes statistics, and more robust inferences based Monte Carlo critical values.

Combined with the Mayoral and Berkes pseudo-long memory tests of the environmental indicators (EF and BC) of each BRICS country, the results provide consistent evidence that only China and Russia have breakpoints in their per capita EF (the China's per capita EF breakpoint occurred in 1990; The per capita EF breakpoint in Russia occurred in 1993). Therefore, a large number of countries follow the evolutionary trend of long memory processes. These breakpoints correspond to the late 1970s and early 1980s. During this period, the Iranian revolution and Iran-Iraq war broke out in 1978–1979. These two major oil producers, accounting for 6% of the world's oil output, experienced a decline in oil products. The real price of oil doubled between 1978 and 1981, which had a negative impact on the economies of BRICS countries where the ecological indicator breakpoints occurred. In response to the oil price shock, BRICS countries' ecological footprints fell significantly, following their economic recessions. We can also see that the two significant structural breakpoints in China correspond to the 1990s, when events, including the Gulf War and coming into force of the Kyoto Protocol at the end of 1997, affected the ecological indicators. However, the break point in the rising process of Russian per capita EF sequence is due to taking the former Soviet Union data as a proxy before 1991, which resulted in sudden shifts in per capita ecological indicators between 1992 and 1995.

Tables 2, 3 summarize the results judged by the estimated values of *d*. The results show the ecological indicators are long memory in all BRICS countries, and the subjects we selected are good for this study. The BRICS are grouped into three categories in term of the degree of long memory. The first group refers to the countries belonging to the ‘mean reversion’ case (i.e., South Africa and Russia in term of CFs, and China, India and South Africa in term of BCs with an order of integration significantly smaller than 1); the second group refers to those countries showing evidence of unit roots (i.e., Brazil and Russia in term of BCs with orders of integration around 1); and the third group refers to countries with divergent ecological indicators (China, India and Brazil in term of CFs with orders of integration significantly above 1).

It is not surprising that the per capita ecological footprints of the vast majority of BRICS countries reported in the empirical results are non-stationary and non-mean-reverting. This reflects that the per capita ecological footprints develop along a trend path, usually along an upward trajectory, and does not return to their means during the sample period. However, due to heavily consuming natural resources, in BRICS countries the ecological carrying capacities evolve along the downward trend. This indicates that these countries have continuously imposed more pressures on the per capita ecological footprints and ecological carrying capacities, resulting in the ecological footprints exceeding the biological carrying capacities of these countries. Therefore these countries uniformly fall into an ecological deficit state, as shown in Figure 2. Some of these countries have high per capita ecological carrying capacities, but have a higher marginal consumption propensity when they are utilizing the nature. For example, Brazil has the highest per capita ecological carrying capacity, but will still faces the dilemma of an ecological deficit due to excessive demand for natural resources.

## Conclusions and Implications

The importance of a composite indicator of environmental degradation (ecological footprint) is that it can go beyond exparte discussions of climate change and sustainability, because it can also track demand for services provided by a wide range of natural resources and ecosystems, rather than just focus on the atmospheric carbon accumulation. In the contemporary world, people have used 1.6 earths to produce the resources consumed and dump the wastes discharged. This trend means that it will take the earth 1 year and 6 months to regenerate natural resources used and absorb wastes per year because of human activities. If this kind of consumption pattern is as of old, the total demand for ecosystems is expected to exceed 80% of natural carrying capacities in another 10 years (39).

Environmental economics is paying more and more attention to the convergence of environmental indicators such as ecological footprints, especially the topics related to natural deterioration. There are two reasons why convergence of environmental indicators is important. (1) The need for close cooperation between countries and regions with similar productivity characteristics when formulating and implementing environmental policies. In order to mitigate the threat of global warming, policies should not be limited to the territorial scope of just one country, but should take actions at the international level. The Tokyo Protocol and the Paris Agreement are examples of the international communities' joint resistance to environmental degradation. (2) The consumption patterns, foreign trade and industrial structure of countries or regions affect the evolving trend of environmental and ecological indicators. Therefore, it is urgent to identify the factors that determine the convergence or divergence of ecological indicators. Environmental policies to protect the nature have an obligation to change the deteriorating trend of environmental indicators. Therefore, identifying the mechanisms that trace the trajectories of changes in ecological indicators is a topic of interest to both policymakers and academic researchers.

This present paper applies the sample data from 1961 to 2017 to study whether or not the stochastic convergence occurred in the ecological footprint and ecological carrying capacity series of BRICS countries. Two rigorous statistical testing methods are employed to identify whether or not the long memory processes (if exist) characterizing ecological indicators are falsely modeled (i.e., identified break points). Through various frequency-domain Whittle estimators and statistical tests, it is found that the fractional integration parameters of the vast majority of per capita EF sequences are greater than 1, indicating that these environmental indicators are non-mean reverting (divergence); The parameters of fractional integration of BC sequences are mostly within the interval 0 < *d* ≤ 1, which indicates that the ecological footprints are non-stationary long-memory processes. The fractional integration parameters of ecological carrying capacities are obviously smaller than those of ecological footprints, but they are still in the non-stationary and long memory domain. The significant discrepancy to the EF indicators in most countries, however, is that the BC sequences are convergent processes, namely mean-reverting. By comparisons, it is found that the convergence of ecological footprints and ecological carrying capacities is closely related to the economic development of various countries. Generally speaking, richer countries have a higher convergence rate of these two indicators being discussed; As the world's largest developing countries, the BRICS countries' ecological indicators are mostly divergent.

From the perspective of existing or potential policies that prevent ecological indicators worsening, the conclusions of this study are very informative. For most BRICS countries, the ecological footprints exhibit non-stationary behaviors, which fully demonstrates that policies that affect the ecological footprints actually have long-term and permanent effects. There are several policies that can address the growing ecological footprints. Such a series of policies includes a carbon tax designed to reduce the demand for carbon and governmental subsidies expended to guarantee the consumption of alternative clean energies. Controls on land use should also be strengthened to protect land, forests and other natural habitats. Regardless of whether or not certain environmental policies are temporary in nature, the fact that the ecological footprints are non-stationary ensures governmental policies can take long-term effects on these indicator's dynamics. China's per capita EF series have undergone structural changes during the sample period, indicating that the rising rate is faster than other BRICS countries. Implications arising from this observation a more stringent and long-term policy tool package should be adopted for energy conservation and carbon emission reduction. The per capita BC sequence in South Africa has also undergone structural changes in the process of persistent decline (judged by the Mayoral test), anticipating a faster decreasing rate. Therefore, in the pursue of economic development, Brazil should get rid of over-exploitating nature and adopt stricter environmental protection policies to prevent the further decline of its ecological carrying capacity.

Because the ecological footprints are found to be non-stationary in most BRICS countries, predicting their evolving dynamics based on past behavior may constitute a great obstacle (if not be feasible). However, their non-stationary behaviors means that policies aimed at changing it will have lasting and persistent impacts. As the vast majority of macroeconomic variables are also non-stationary, especially for level values (not differenced values), the ecological footprints may have the same fractional integration order as that of macroeconomic variables, thus indicating the existence of cointegration between these variables, in other words, long-term equilibrium relationship, which can assist in formulating relevant environmental policies.

Convergence of environmental indicators between countries is considered as a fair share of carbon dioxide emission rights. However, different countries have different natural endowments, and furthermore resources can be transferred between different countries. The stationarity of a sequence provides information about whether the effects of a shock to the sequence is temporary or permanent. A stationary sequence manifest itself that the effects of this shock are temporary. Therefore, if the impacts of shocks to the ecological footprint sequences dissipate quickly, these sequences of various countries converge to the sample means stochastically. A non-stationary sequence indicates that the effects of the shock hitting it are permanent, i.e. this sequence will deviate from the sample mean eventually. Similarly, when a ecological indicator is non-stationary, environmental policies that affect it have persistent, and even permanent effects. At the same time, the convergence of ecological indicators between countries shows that it is necessary to adopt internationally cooperative policies to protect the vulnerable environment. The lack of convergence indicates that these policies are out of permanent effects and the cross-broad policies are not very powerful.

Due to the long-memory characteristics of environmental indicators, the impacts of a positive shock (usually comes from an environmental policy) will be long-term and this policy effect will be better. For example, specific policies include energy efficiency improvement projects and subsidies for renewable energies. However, the divergence of environmental indicators is more disadvantageous. If an adverse random impact occurs, the impact will be very long-term, and a stronger policy intervention is needed to reverse the trajectory of environmental deterioration. In order to deter the worsening trend of ecological indicators, a steadfast policy stance is necessary. Therefore, we argue that: (1) A policy that is designed to be temporary or eventually revoked cannot expect to have lasting effects. (2) A policy that is either permanent or believed to be permanent will often have stronger and more persistent effects. Based on our empirical results, the degree of commitment is as important or more important than the validity or the potential effects of a policy.

In addition, some country-specific characteristics, such as the degree of economic development, industrial structure and consumption patterns, should also be taken into account. As high-income countries and developed countries formulate some better policies and more effective measures to confront the challenge of the ecological indicator worsening, low-income countries should borrow some advanced experiences of developed countries who share similar evolving trajectories of ecological indicator to achieve greater progress in curbing the deterioration of ecological indicators.

## Data Availability Statement

The original contributions presented in the study are included in the article/supplementary material, further inquiries can be directed to the corresponding author/s.

## Author Contributions

The author confirms being the sole contributor of this work and has approved it for publication.

## Funding

We acknowledge financial support from the research project A Research on Parameter Identification and Application of DSGE Models Allowing for Indeterminacy (Approval Number: 71863008) sponsored by National Natural Science Foundation of China.

## Conflict of Interest

The author declares that the research was conducted in the absence of any commercial or financial relationships that could be construed as a potential conflict of interest.

## Publisher's Note

All claims expressed in this article are solely those of the authors and do not necessarily represent those of their affiliated organizations, or those of the publisher, the editors and the reviewers. Any product that may be evaluated in this article, or claim that may be made by its manufacturer, is not guaranteed or endorsed by the publisher.
